# Pharmacological and behavioral investigation of putative self-medicative plants in Budongo chimpanzee diets

**DOI:** 10.1371/journal.pone.0305219

**Published:** 2024-06-20

**Authors:** Elodie Freymann, Susana Carvalho, Leif A. Garbe, Dinda Dwi Ghazhelia, Catherine Hobaiter, Michael A. Huffman, Geresomu Muhumuza, Lena Schulz, Daniel Sempebwa, Florian Wald, Eguma R. Yikii, Klaus Zuberbühler, Fabien Schultz

**Affiliations:** 1 Primate Models for Behavioural Evolution Lab, Institute of Human Sciences, Department of Anthropology and Museum Ethnography, University of Oxford, Oxford, United Kingdom; 2 Gorongosa National Park, Sofala, Mozambique; 3 Interdisciplinary Centre for Archaeology and the Evolution of Human Behaviour, University of Algarve, Faro, Portugal; 4 Ethnopharmacology & Zoopharmacognosy Research Group, Department of Agriculture and Food Sciences, Neubrandenburg University of Applied Sciences, Neubrandenburg, Germany; 5 ZELT–Center for Nutrition and Food Technology gGmbH; 6 Wild Minds Lab, School of Psychology and Neuroscience, University of St Andrews, St Andrews, United Kingdom; 7 Budongo Conservation Field Station, Masindi, Uganda; 8 Wildlife Research Center, Inuyama Campus, Kyoto University, Inuyama, Japan; 9 Czech University of Life Sciences Prague, Prague, Czech Republic; 10 Department of Comparative Cognition, Institute of Biology, University of Neuchâtel, Neuchâtel, Switzerland; 11 Pharmacognosy and Phytotherapy, School of Pharmacy, University College of London, London, United Kingdom; University of Buea, CAMEROON

## Abstract

Wild chimpanzees consume a variety of plants to meet their dietary needs and maintain wellbeing. While some plants have obvious value, others are nutritionally poor and/or contain bioactive toxins which make ingestion costly. In some cases, these nutrient-poor resources are speculated to be medicinal, thought to help individuals combat illness. In this study, we observed two habituated chimpanzee communities living in the Budongo Forest, Uganda, and collected 17 botanical samples associated with putative self-medication behaviors (e.g., bark feeding, dead wood eating, and pith-stripping) or events (e.g., when consumer had elevated parasite load, abnormal urinalysis, or injury). In total, we selected plant parts from 13 species (nine trees and four herbaceous plants). Three extracts of different polarities were produced from each sample using *n*-hexane, ethyl acetate, and methanol/water (9/1, *v/v*) and introduced to antibacterial and anti-inflammatory *in vitro* models. Extracts were evaluated for growth inhibition against a panel of multidrug-resistant clinical isolates of bacteria, including ESKAPE strains and cyclooxygenase-2 (COX-2) inhibition activity. Pharmacological results suggest that Budongo chimpanzees consume several species with potent medicinal properties. In the antibacterial library screen, 45 out of 53 extracts (88%) exhibited ≥40% inhibition at a concentration of 256 μg/mL. Of these active extracts, 41 (91%) showed activity at ≤256μg/mL in subsequent dose-response antibacterial experiments. The strongest antibacterial activity was achieved by the *n-*hexane extract of *Alstonia boonei* dead wood against *Staphylococcus aureus* (IC50: 16 μg/mL; MIC: 32 μg/mL) and *Enterococcus faecium* (IC50: 16 μg/mL; MIC: >256 μg/mL) and by the methanol-water extract of *Khaya anthotheca* bark and resin against *E*. *faecium* (IC50: 16 μg/mL; MIC: 32 μg/mL) and pathogenic *Escherichia coli* (IC50: 16 μg/mL; MIC: 256 μg/mL). We observed ingestion of both these species by highly parasitized individuals. *K*. *anthotheca* bark and resin were also targeted by individuals with indicators of infection and injuries. All plant species negatively affected growth of *E*. *coli*. In the anti-inflammatory COX-2 inhibition library screen, 17 out of 51 tested extracts (33%) showed ≥50% COX-2 inhibition at a concentration of 5 μg/mL. Several extracts also exhibited anti-inflammatory effects in COX-2 dose-response experiments. The *K*. *anthotheca* bark and resin methanol-water extract showed the most potent effects (IC50: 0.55 μg/mL), followed by the fern *Christella parasitica* methanol-water extract (IC50: 0.81 μg/mL). This fern species was consumed by an injured individual, a feeding behavior documented only once before in this population. These results, integrated with associated observations from eight months of behavioral data, provide further evidence for the presence of self-medicative resources in wild chimpanzee diets. This study addresses the challenge of distinguishing preventative medicinal food consumption from therapeutic self-medication by integrating pharmacological, observational, and health monitoring data—an essential interdisciplinary approach for advancing the field of zoopharmacognosy.

## Introduction

‘Medicinal foods’ refer to resources in the diet that have potential curative value due to the presence of plant secondary metabolites (PSMs) [1, 2]. PSMs are compounds that usually occur only in special, differentiated cells [3] and which help plants defend against predators, pathogens, and competitors [4–7]. PSMs can have a range of functions, including the inhibition of microbial, fungal, and competitor growth [8]. While some PSMs can be toxic at high doses, these compounds can also promote the health of human and non-human consumers [8–10]. Research suggests 15–25% of primate and other mammalian diets consist of medicinal foods [9, 11]. These resources likely play a critical role in animal health-maintenance by passively preventing or reducing the impact of parasitic infections or other pathogens [9–14].

While most animals likely consume foods with medicinal properties as part of their normal diets, fewer species have been shown to engage in therapeutic self-medication. Huffman [15] defines this type of self-medicative behavior as the active extraction and ingestion, by an ill individual, of medicinal resources with little nutritional value. Instead of an individual passively benefiting from a plant’s medicinal properties through normal feeding, this form of self-medication requires basic awareness of the resource’s healing properties. One of the best-studied animals to engage in this form of self-medication is our closest living relative: the chimpanzee.

Wild chimpanzees (*Pan troglodytes*), across at least sixteen field sites [15] have demonstrated therapeutic self-medication using two well-established self-medicative behaviors: leaf swallowing [16, 17] and bitter-pith chewing [18]. Leaf swallowing, first reported by Wrangham [19, 20] and described by Wrangham & Nishida [21], involves the careful selection and ingestion of whole, hispid leaves. This behavior was later demonstrated to expel internal parasites (i.e. *Oesophagostomum* sp. and *Bertiella studeri*) from the gut [16, 17, 22, 23]. The functional mechanism responsible for this anthelminthic effect is considered to be primarily “mechanical” [9] as, rather than a chemical compound, the leaf’s indigestibility, brought about by the trichomes on its surface—stimulates gut motility in the swallower [17, 23, 24].

The second established behavior is bitter-pith chewing, which involves the stripping of outer bark and leaves from the soft new stem growth of the shrub, *Vernonia amygdalina*, exposing the inner pith. Individuals chew the pith and ingest only the bitter juices while spitting out the fibers [18, 25]. Bitter-pith chewing is considered ‘phytochemical’ self-medication [9], as its anthelminthic effect appears to be the result of bioactive PSMs [26–29]. This behavior’s medicinal effect was associated with a significant drop in the infection intensity of *Oesophagostomum stephanostomum* nematodes [25], suggesting that the bitter compounds directly affect the adult worms. This hypothesis was supported by *in vivo* studies conducted by Jisaka et al. [30], demonstrating that extracts from the pith permanently paralyzed adult Schistosome parasites. *V*. *amygdalina* is also used to aid gastrointestinal discomfort and other signs of parasitosis in humans and livestock, symptoms also displayed by chimpanzees ingesting the plant’s bitter pith [9, 18, 25, 31]. The bitter piths of other plant species are reported to be chewed by chimpanzees across field sites but detailed studies on their medicinal properties have yet to be conducted [9].

Beyond these two established behaviors, not much is known about the phytochemical self-medicative repertoires of wild chimpanzees, although some behaviors associated with the ingestion of specific plant parts or processing techniques have been recommended for further investigation [9, 15, 32]. One of these behaviors is bark feeding, which involves the ingestion of living stem bark and/or cambium [33], and which has been observed in at least eleven established field sites [33–43]. Bark feeding has been suggested as a medicinal behavior in chimpanzees and other primates, used to aid in the chemical control of intestinal nematode infection and to relieve gastrointestinal upset [9]. Bark is characteristically highly fibrous, heavily lignified, sometimes toxic, relatively indigestible, and nutrient-poor [44]. However, the contribution of bark in chimpanzee diets and toward general health is still poorly understood [though see: 45]. In this study, the bark of eight species ingested by Budongo chimpanzees (*Scutia myrtina*, *Cynometra alexandri*, *Alstonia boonei*, *Ficus exasperata*, *Ficus variifolia*, *Syzygium guineense*, *Desplatsia dewevrei*, *Khaya anthotheca)* was screened for antibiotic and anti-inflammatory properties, to better understand the function of bark feeding behaviors and the role this behavior may play in the health maintenance of chimpanzees. For the species *K*. *anthotheca*, we tested a mixture of bark and congealed resin, which Budongo chimpanzees were observed to particularly target throughout the study period.

Another putative self-medicative behavior is dead wood eating [9, 35], which involves the consumption of decomposing cambium from dead trees. To date, the majority of studies examining this behavior in apes have focused on exploring potential mineral and nutritional benefits, rather than investigating pharmacological properties [46–49]. Many of these studies suggest that dead wood is exploited by chimpanzees as a source of sodium in environments where this mineral is otherwise scarce [48, 49]. Our study evaluates the pharmacology of two species of dead wood (*A*. *boonei* and *Cleistopholis patens)* consumed by the Sonso community of chimpanzees to determine whether this behavior may have multiple functions or health benefits.

The ingestion of pith material from other species has also been suggested as putatively self-medicative [34, 50, 51]. However, unlike *V*. *amygdalina* bitter-pith, some of these plant piths appear bland or tasteless. While Wrangham et al. have previously suggested that pith is likely a high-fiber fallback food [52], De la Fuente et al. review several pith species targeted by chimpanzees with proposed medicinal properties [32]. In our study, two species of non-bitter piths (*Marantachloa leucantha* and *Acanthus polystachyus)*, were collected for pharmacological assessment. *M*. *leucantha* was observed on several occasions being stripped, masticated, and spat out after the juice was extracted from the pith, whereas *A*. *polystachyus* was observed being stripped, masticated, and swallowed. Both of these species are also ingested by chimpanzees in Kibale National Park, Uganda [52].

Establishing phytochemical self-medicative behaviors in wild animals is difficult and time consuming, as the burden of proof is high, self-medicative events can be rare relative to other behaviors, and methods often require multidisciplinary expertise and collaboration [9]. Past studies have utilized ethnopharmacological methods to determine specific medicinal properties of foods consumed by primates [11], greatly advancing our understanding of the relationship between primate diets and health. However, a key challenge for establishing novel self-medicative behaviors is differentiating between medicinal food consumption and therapeutic self-medication. While pharmacological data interpreted on its own is crucial for establishing the presence of medicinal resources in chimpanzee diets, the integration of observational and health monitoring data is needed to parse therapeutic self-medicative behaviors from normal feeding behaviors with inadvertent health benefits. Furthermore, the importance of collecting *in situ* samples from the locations where putative self-medicative behaviors are observed is paramount, as ecological, climatic, and anthropogenic variables can cause variation in the bioactivity of plants across habitats [53].

In total, we investigated the bioactivity of 51 plant extracts produced from 17 part-specific samples (across 13 species), collected in the Budongo Forest. Each extract was tested for inhibition of bacterial growth as well as anti-inflammatory COX-2 inhibition activity. Due to limitations in scope, funding, and the unavailability of anthelminthic assays for wild animal parasites, none were not conducted in this study, restricting specific identification of parasiticidal behaviors. Assay results are reported and contextualized in this study with direct behavioral evidence and health monitoring data.

## Materials and method

### Study site and subjects

Behavioral data, health monitoring metrics, and botanical samples were collected from the Budongo Central Forest Reserve in Uganda (1°35′– 1°55′ N, 31°18′–31°42′ E). An overview of methodological workflow can be found in **S2 Fig**. The Budongo Conservation Field Station (BCFS) site, founded in 1990, is composed of continuous, semi-deciduous forest and contains two habituated Eastern chimpanzee (*Pan troglodytes schweinfurthii*) communities [54]. The Sonso community has been studied continuously since 1992, and the ages, social relationships, demographics, and diet of its members are well documented [55, 56]. The Sonso population was ~68 individuals at the time of data collection, and the home range covered an area of ~5.33 km^2^ [57]. Waibira, a larger group of at least 105 individuals, was more recently habituated, with consistent data collection beginning in 2011. The Waibira maximum home range area was ~10.28 km^2^ [57].

### Behavioral data collection

All samples were collected in the Budongo Forest within the Sonso home range, based on behavioral observations from the study period and supporting evidence from the site’s long-term data of their use. Behavioral and health data were collected from two neighboring chimpanzee communities, each for one four-month field season (Sonso: June-October 2021, Waibira: June-October 2022). Data collected between June-September 2021 informed subsequent plant sample collection for pharmacological analysis, which occurred in early September 2021. Behavioral data collected after sample collection provided additional behavioral context for ingestion of these species. Behavioral data were collected between 07:00 and 16:30 in Sonso and between 06:30 and 17:00 in Waibira using day-long focal animal follows *sensu* Altman et al. [58]. This data was recorded using Animal Observer (AO) on iPad and *ad libitum* feeding events were recorded for any unusual feeding behaviors, including but not limited to bark ingestion, dead wood eating, pith stripping, and geophagy. All feeding events were filmed on a Sony Handycam CX250. We prioritized focal follows on individuals with wounds, high or diverse parasite loads identified through on-going monitoring, or known ailments. However, consecutive day follows of priority individuals were not always possible—or were avoided when they might contribute to increased stress in particularly vulnerable individuals. Throughout the study, using this protocol, 27 Sonso individuals (♂:11; ♀:16) and 24 Waibira individuals (♂:14; ♀:10) were observed. Authors collecting behavioral data were blind to pharmacological results during both study periods.

### Health monitoring

Individual health data were recorded in both communities, including opportunistic macroscopic and microscopic fecal analysis and urinalysis testing. While anthelminthic assays were not run in this study, parasite load was opportunistically assessed to provide additional health context for each observation. As the presence of certain helminths may impair a host’s immunological response to bacterial, viral, and protozoal pathogens [59], parasite load can provide a proxy measurement for overall health. Similarly, a reduced immune system and increased stress caused by co-infections could render a host more susceptible to virulent endoparasites [60, 61]. When helminths and/or proglottids were found in samples, they were collected and preserved in ethanol for later identification. To quantify parasite loads, fecal samples were analyzed using the McMaster Method [9, 25, 62]. Urinalysis samples were taken opportunistically using multi-reagent Urine Dipstick Test 9-RC for Urotron RL9 to assess the health and physiological status of group members following methods established by Kaur & Huffman [63]. Urinalysis metrics considered in this study included: **leukocytes** (LEU) associated with pyuria caused by UTI, balanitis, urethritis, tuberculosis, bladder tumors, viral infections, nephrolithiasis, foreign bodies, exercise, glomerulonephritis, and corticosteroid and cyclophosphamide use; **blood** (BLO) associated with peroxidase activity of erythrocytes, and UTIs; and **ketones** (KET) associated with pregnancy, carbohydrate-free diets, starvation, and diabetes [64]. Test results were interpreted *in situ* using a colorimetric scale. We considered a result ‘abnormal’ if the colorimetric scale indicated a positive result when the expected result was negative or if the result was outside the specified test parameters according to the manufacturer.

### Plant sample selection for bioactivity testing

Plants were selected for pharmacological testing after three months of data collection in the Sonso community. We selected 10 samples (from 9 species) based on direct observations during this period. These observations included individuals targeting plant parts associated with putative self-medicative behaviors (i.e., bark feeding, dead wood eating, pith-stripping) or sick/wounded individuals seeking out unusually consumed resources. We then selected an additional five species, the ingestion of which had not been directly observed, for testing based on their historical inclusion in Sonso chimpanzees’ bark feeding repertoire. GM, who has worked at the field station for over thirty-years, has previously observed bark feeding on each of these selected species. These historic observations enabled collection of bark samples from specific trees known to have been previously stripped. In two cases, leaf samples were collected from tree species that were also selected for bark samples (*S*. *guineense* and *F*. *exasperata)*. While neither Sonso nor Waibira chimpanzees have been observed ingesting the leaves of *S*. *guineense*, a sample was collected to enable comparison of bioactivity across plant parts. *F*. *exasperata* leaves are consumed in both communities; however, we found no behavioral evidence for use in unusual contexts. In some cases, direct observation of an event involving one of the collected species occurred after botanical collection was complete. These *post hoc* behavioral observations are reported in this paper, although they did not impact sample selection.

### Collection of sample material

Plants were collected from the Sonso community home range following best practice procedures [65], using sustainable harvesting methods [66]. See **S1 File** for more information. Voucher accession numbers are reported in **Table 3**. Digital images of voucher specimens can be found in **S3 Fig**. The currently recognized scientific names of each species were confirmed on https://mpns.science.kew.org/. Plant family assignments were done in accordance with The Angiosperm Phylogeny Group IV guidance [67].

### Ethnobotanical literature review

We conducted a post-hoc ethnomedicinal review of all species collected for this study using Google Scholar, PROTA, and Kokwaro’s ethnomedicinal pharmacopeia [68]. To search databases, we used scientific names and synonyms for each plant as keywords [65].

### Plant processing and extractions

At Neubrandenburg University of Applied Sciences, samples were ground using a food processor. Extractions were produced using two solvents and a solvent mixture (*n*-hexane, ethyl acetate, and methanol/water (*v/v* 9/1)), allowing for the selective isolation of components with varying solubilities and polarities. Methanol-water, the solvent with the highest polarity, generally extracts primary plant metabolites (e.g., polar compounds such as proteins, amino acids, and carbohydrates). Nonpolar solvents like *n-*hexane extract nonpolar compounds like lipids, making n-hexane a preferred solvent for oil or wax extraction. Extractions with each solvent were achieved through double maceration of new material (non-successively). Extraction suspensions were placed on a shaker at 80 rpm at room temperature for minimum 72h, followed by vacuum filtration. Processes were repeated with the leached material. Filtrates were then combined and dried using a vacuum evaporator, labeled, and stored at -20°C until needed for assays.

### Sample solution preparation

To create sample solutions, each crude extract was dissolved in DMSO (Carl Roth) at a concentration of 10 mg/mL. To ensure a homogenous solution, samples were mixed with a vortex mixer and, if necessary, treated with sonication at room temperature or up to 55°C for samples with low solubility. Each extract solution was then tested for inhibition of bacterial growth as well as anti-inflammatory COX-2 inhibition activity. Solutions were stored at -20°C when not in use.

### Antibacterial susceptibility tests

#### a. Bacterial strains

For antibacterial assays, eleven multidrug-resistant clinical isolate strains from nine species were used. This process increased the study’s applicability for early-stage drug discovery, specifically relevant to the threat of antimicrobial resistance (AMR). Seven of these strains (from six species) are classified as ESKAPE pathogens, including *Enterococcus faecium* (DSM 13590), *Staphylococcus aureus* (DSM 1104; DSM 18827), *Klebsiella pneumoniae* (DSM 16609), *Acinetobacter baumannii* (DSM 102929), *Pseudomonas aeruginosa* (DSM 1117), and *Enterobacter cloacae* (DSM 30054), meaning they are highly virulent and resistant to antibiotics [69]. A strain of the foodborne pathogen *Escherichia coli* (DSM 498) with AMR as well as a non-resistant *E*. *coli* strain (DSM 1576) were also included in the study. Although not an ESKAPE pathogen, *E*. *coli* is widely known for causing bacterial diarrhea and AMR strains are a major cause of urinary tract infections [70, 71]. Strains of *Stenotrophomonas maltophilia* (DSM 50170) and *Salmonella enterica* subsp. *enterica* (DSM 11320) were also tested. More information on specific clinical isolates/strains, their individual resistance profiles, and antibiotics used can be found in the **S5 & S6 Tables in S2 File.** Clinical and Laboratory Standards Institute (CLSI) guidelines for broth microdilution testing (M100-S23) were followed [72].

#### b. Growth inhibition screening and dose-response study

The broth dilution *in vitro* methods for bacterial susceptibility assessment have previously been described by Schultz et al. [69]. The standardized bacterial working cultures were pipetted into sterile 96-well microtiter plates (Greiner Bio-One International, CELLSTAR 655185). Extracts and antibiotic (64–1 μg/mL), vehicle and sterility controls, were then added into respective wells. Initial optical density measurement (600 nm) was performed, accounting for absorbance of extracts. Plates were incubated at 37°C for 18 h, except for *A*. *baumannii* which was incubated for 22h in accordance with strain characteristics (**S5 Table in S2 File)**. After incubation, a final optical density reading (600 nm) was conducted. Percent inhibition values were calculated and the IC_50_ and MIC values were determined [69, 73]. The IC_50_ value is defined as the lowest concentration at which an extract showed ≥ 50% inhibition, and the MIC is the lowest concentration at which an extract displayed ≥ 90% inhibition. A total of 51 samples underwent single-dose pre-screening for growth inhibition (in triplicate) at the concentration of 256 μg/mL on eleven pathogens. Samples showing ≥40% growth inhibition were further tested in a dose-response study with two-fold serial dilution at descending concentrations from 256 to 4 μg/mL. The dose-response experiments were done as biological replicates on separate days in triplicate (technical replicates) to validate reproducibility. Positive controls (antibiotics) and negative controls (vehicle control and sterile media control) were always included. Further details on bacteria standardization can be found in **S1 File**. Information on plate setup for bacterial library screens and dose-response assays can be found in **S4 Fig**.

### COX-2 inhibition assay

Anti-inflammatory assays were assessed using an *in vitro* COX inhibitor screening assay kit (Cayman Item No: 701080), with modifications previously described in Schultz et al. [74]. All extracts were first screened in duplicate for inhibition against human recombinant COX-2 at an initial concentration of 50 μg/mL. For extracts exhibiting at least 50% inhibition, the concentration was then lowered to 10 μg/mL, 5 μg/mL, and 2.5 μg/mL. The most active extracts were taken to dose-response experiments for determination of IC_50_ values (**Table 5**). The assay was done in two steps: 1) the COX reaction step in which the prostaglandin H_2_ (PG) was produced (which was further reduced to the more stable prostaglandin F_2α_ by addition of stannous chloride), and 2) an acetyl choline esterase competitive ELISA step to quantify the produced prostaglandin and calculate a potential enzyme inhibition caused by the extracts. The pure compound and selective COX-2 inhibitor DuP-769 was included as a positive control. DMSO was included as the vehicle control for determining 100% enzyme activity. Information on ELISA plate setup for anti-inflammation assays can be found in **S5 Fig**.

### Ethics statements

Behavioral data used in this study were collected with the approval of the Uganda Wildlife Authority (permit #: COD/96/05) and the Uganda National Council for Science and Technology (permit #: NS257ES). Exportation of samples for pharmacological testing were conducted under UNCST permit #: NS104ES. Behavioral data collection adhered to International Primatological Society’s Code of Best Practice for Field Primatology [75]. No exported samples were listed under CITES. Plant samples were exported in collaboration with Makerere University (permit #: UQIS00005033/93/PC), issued by the Ugandan government, and transported to Neubrandenburg University of Applied Sciences in accordance with the Nagoya Protocol. A CUREC was approved by the University of Oxford (Ref No.: SAME_C1A_22_080). The authors report no conflict of interest.

## Results

### Behavioral observations

Several unusual feeding events and putative self-medicative behaviors were recorded over 116 total field days. **Table 1** reports all species collected for pharmacological testing and provides behavioral justifications for collection. Images from some of these events can be found in **S1 Fig**.

**Table 1:** Relevant behavioral observations associated with plant species selected for pharmacological screening.

Species	Plant part tested & associated behavior	Previously proposed as self-medicative resource^1^	Previous reports of ingestion^2^		Justification for collection / Notable observation(s) from study period	
			Sonso	Waibira	Sonso (Observed before sample selection)	Waibira (Observed after sample selection)
*Acanthus polystachyus* Delile.	**Pith** (Pith Stripping)	Yes	✓	✓	***Species collected based on known inclusion in Sonso’s pith-stripping repertoire*.** **Case 1:** On 8/13/2021, sub-adult male (KC) stripped pith ~2h before leaf-swallowing. **Case 2:** On 8/26/2021, juvenile male (MZ) stripped pith on the same day he consumed several other putative self-medicative resources (**see Table 1: *S*. *myrtina*, Case 1**).	**Case 1:** On 9/13/2022, a large group travelled into the Sonso core area to strip pith, despite proximity of vocalizing Sonso males. Waibira group travelled directly to patch of *A*. *polystachius*, consumed pith for ~30 minutes, and returned directly to Waibira’s core area.
*Alstonia boonei* De Wild.	**Stem Bark** (Bark Feeding)	Yes	✓	✓	***Species collected based on known inclusion in Sonso’s bark ingestion repertoire*.** ***No direct observations*.**	***No direct observations*.**
	**Dead Wood** (Dead Wood Eating)	No	✓	✓	***Species collected based on known inclusion in Sonso’s dead wood eating repertoire*.** **Case 1:** Adult male (SM), sub-adult male (MB), and juvenile male (MZ) consumed dead wood from decaying, standing trunk on 10/6/2021. Event occurred while community was outside core area. Healing bite marks indicated previous visit(s) to the tree. MB was observed with diarrhea two days before, shedding proglottids of *Bertiella* sp., and harboring unidentified protozoa. Four days after the event, SM was found to have *Ascaris* (50 EPG*)*, *Ancylostoma* (50) *Oesophagostomum* (250 EPG), and *Trichostrongyloides* (100 EPG) eggs in his feces.	***No direct observations*.**
*Cleistopholis patens* (Benth.) Engl. & Diels.	**Dead Wood** (Dead Wood Eating)	No	✓	✓	***Species collected based on known inclusion in Sonso’s dead wood eating repertoire*.** **Case 1:** On two occasions, nine days apart, adult male (ZL) consumed dead wood. On first occasion, 7/24/2021, ZL travelled away from the group with a juvenile, orphaned male (OZ) to eat dead wood. A fecal analysis collected from ZL two days later showed the presence of *Ascaris* sp. (7600 EPG), *Ancylostoma* (50 EPG), *Oesophagostomum* (1050 EPG), a segment of *Taenia*, and *Strongyloide* larvae. **Case 2:** On 8/2/2021, ZL again travelled away from the group while on inter-community patrol to eat dead wood, accompanied by a different juvenile, orphan male (KJ). ZL’s feces from this day had *Ascaris* (50 EPG), *Ancylostoma* (50 EPG), *Oesophagostomum* (1200 EPG), *Strongyloides* (100 EPG), *Trichostrongyloides* (50 EPG). A urine test from this day found ZL positive for leukocytes. **Case 3:** An adult female (KL) and her two offspring broke-off from a group on 9/16/2021 to eat dead wood for up to 20 minutes. They were joined by adult female (DR). KL had severe diarrhea immediately following the bout and was found to have *Ancylostoma* (300 EPG) in her feces. **Case 4:** On 8/26/21, juvenile male (MZ) was observed eating dead wood on day he consumed several other putative self-medicative resources (**see Table 1: *S*. *myrtina*, Case 1**).	***No direct observations*.**
*Christella parasitica* (L.) H. Lév.	**Fern** (Feeding)	No	✓	×	***Species collected based on unusual feeding events*.** **Case 1:** Adult male (PS) ate leaves of *C*. *parasitica* on 8/16/2021 with a newly injured hand while travelling outside core area. PS was the only individual in a large group to seek out and subsequently feed on ferns. No parasitological or urinalysis data are available for PS on this day.	***No direct observations*.**
*Cynometra alexandri* C. H. Wright.	**Stem Bark** (Bark Feeding)	Yes	✓	✓	***Species collected based on known inclusion in Sonso’s bark feeding repertoire*.** ***No direct observations*.**	**Case 1:** On 9/2/2022, adult male (SAM) was observed ingesting bark in a group feeding bout while coughing and sneezing. His symptoms improved in the following days. SAM’s fecal analysis from this day contained *Ancylostoma* (50 EPG), *Oesophagostomum* (200 EPG), and *Trichuris* (100 EPG). **Case 2:** On 9/2/2021, during the same bark feeding event as **Case 1**, adult female (ELD) was observed bark feeding while in estrus, the period of sexual receptivity in female chimpanzees marked by physical and behavioral changes conducive to mating. Her fecal sample contained *Ascaris* (550 EPG), *Ancylostoma* (50 EPG), *Oesophagostomum* (250 EPG), *Strongyloides* (150 EPG), *Trichuris* (50 EPG), and Unidentified cestode eggs (500 EPG). **Case 3:** On 9/30/2022, adult male (ALF) was seen with a wounded mouth bark stripping with a group of males. **Case 4:** On 9/24/2022, sub-adult male (JNO) was observed bark stripping alone. JNO’s fecal analysis from this day had *Oesophagostomum* (800 EPG).
*Desplatsia dewevrei* (De Wild. & T. Durand) Burret.	**Stem Bark** (Bark Feeding)	No	✓	×	***Species collected based on known inclusion in Sonso’s bark feeding repertoire*.** ***No direct observations*.**	***No direct observations*.**
*Ficus exasperata* Vahl.	**Stem Bark** (Bark Feeding)	Yes	✓	✓	***Species collected based on known inclusion in Sonso’s bark feeding repertoire*.** **Case 1:** On 7/13/2021, while Sonso was on inter-community patrol in Waibira, two sub-adult males (ZD, MB) and one juvenile male (MZ) stripped bark for ~5-minutes. No health data is available from any of these individuals.	***No direct observations*.**
	**Mature Leaves** (Feeding)	Yes	✓	✓	***Plant part selected for cross-plant bioactivity comparison*.** ***Multiple direct observations*.**	***Multiple direct observations*.**
*Ficus variifolia* Warb.	**Stem Bark** (Bark Feeding)	No	✓	×	***Species collected based on known inclusion in Sonso’s bark feeding repertoire*.** ***No direct observations*.**	***No direct observations*.**
*Khaya anthotheca* (Welw.) C. DC.	**Stem Bark & Resin** (Bark Feeding)	Yes	✓	✓	***Species collected based on known inclusion in Sonso’s bark feeding repertoire*.** **Case 1:** Adult male (PS) fed on bark and resin on 10/6/2021 while the rest of the group waited on the ground. His fecal sample had *Ascaris* (100 EPG), *Ancylostoma* (700 EPG), *Oesophagostomum* (1750 EPG), *Strongyloides* (50 EPG) and a *Bertiella* sp. proglottid. PS also had a new wound on his arm which he groomed throughout the day. Wounded individuals were observed consuming bark and resin of this species on at least two other occasions. **Case 2:** On 7/15/2021, a urinalysis test from adult female (IN) tested positive for leukocytes following ingestion of bark and resin. IN had been observed with severe diarrhea the previous day. **Case 3:** On 7/8/2021, adult female (WM) with severe diarrhea tested positive for leukocytes and trace levels of blood on urinalysis test following ingestion of bark and resin. **Case 4:** On 8/9/2022, a juvenile female (DB) with a persistent cough consumed bark and resin. **Case 5:** On 8/26/2021, juvenile male (MZ) ate bark and resin with several other putative self-medicative resources tested in this study (**see Table 1: *S*. *myrtina*, Case 1**). **Case 6:** On 8/13/21, adult female (KL) ate bark and resin a few hours before leaf-swallowing.	***No direct observations*.**
*Marantochloa leucantha* (K.Schum.) Milne-Redh.	**Pith** (Pith Stripping)	No	✓	✓	***Species collected based on known inclusion in Sonso’s pith-stripping repertoire*.** **Case 1:** On 9/13/2021, juvenile male (MZ) stripped pith with two unrelated adult females (NB and WM). Fecal analysis from MZ revealed presence of *Ancylostoma* (650 EPG), *Oesophagostomum* sp. (600 EPG), and *Ascaris* sp. (50 EPG).	**Case 1:** On 7/15/2022, adult female (BAH) stripped and wadged pith while her juvenile son (BRI) rested. BAH’s urinalysis showed high ketone levels. Her fecal sample had *Ascaris* (50 EPG), *Ancylostoma* (50 EPG), *Oesophagostomum* (650 EPG), and a *Taenia* segment. **Case 2:** Between 7/27/2022-7/31/2022, adult male (FID) stripped pith three times in a four-day period. FID’s fecal samples from ten and five days before the first event both had *Trichuris* sp. (whipworm), a nematode known to cause health complications. On the first day, FID was the only one to strip pith despite proximity of another adult male (MAC). The bout lasted up to 20 minutes. The following day, FID stripped pith alone, and later that day stripped *Ficus saussureana* bark with two other sub-adult males, both of whom had expelled *Bertiella* sp. proglottids within four days of the event. Two days later, FID stripped *M*. *leucantha* again, alone. A fecal sample collected from FID the day after the final event, contained eggs of *Ancylostoma* sp. (200 EPG), *Oesophagostomum* (100 EPG), *Strongyloides* (150 EPG), and *Trichostrongyloides* (50 EPG). No *Trichuris* eggs were detected in the final sample.
*Scutia myrtina* (Burm.f.) Kurz.	**Stem Bark** (Bark Strip)	No	×	×	***Species collected based on unusual feeding events*.** **Case 1:** Juvenile male (MZ) travelled a far distance from the main group with sub-adult brother (MB) on 8/26/2021, and both stripped bark in periphery of home range. On the same day, the individuals were also observed consuming several other putative medicinal resources, including *A*. *polystachyus* pith, *C*. *patens* dead wood, and *K*. *anthotheca* bark and resin, all of which were tested in this study. MZ whimpered consistently throughout the day and his fecal sample taken during the event showed six species of internal parasites including *Ascaris* (50 EPG), *Trichuris* (50 EPG), *Taenia* (50 EPG), *Strongyloides* (200 EPG), *Oesophagostomum* (250 EPG), and *Ancylostoma* (100 EPG). Plant showed evidence of previous stripping. Fresh sample was cut from plant near where bark was stripped.	***No direct observations*.**
	**Stripped Stem Bark Refuse** (Bark Strip)	No	×	×	***Species collected based on unusual feeding events*.** **Case 1:** During event mentioned above, stripped bark was discarded by individuals during processing. A sample was collected from the ground to assess potential differences between consumed and discarded bark.	***No direct observations*.**
*Syzygium guineense* (Willd.) DC.	**Stem Bark** (Bark Feeding)	No	✓	×	***Species collected based on known inclusion in Sonso’s bark feeding repertoire*.** ***No direct observations*.**	***No direct observations*.**
	**Mature Leaves** (n. a.)	Yes	×	×	***Plant part selected for cross-plant bioactivity comparison*.** ***No direct observations*.**	***No direct observations*.**
*Whitefelida elongata* (P. Beauv.) De Wild. & T. Durand.	**Young Leaves** (Feeding)	No	✓	✓	***Species collected based on unusual feeding events*.** **Case 1:** On 8/16/21, adult male (PS) with severely injured hand ate these leaves immediately before ingesting *C*. *parasitica* (fern) (**See Table 1: *C*. *parasitica*, Case 1**). **Case 2:** On 8/25/2021, juvenile male (MZ) ate these leaves twice throughout the day. His older brother (MB), who he travelled with throughout the day, did not eat them. The following day, MZ was reported eating several putative self-medicative resources tested in this study (**See Table 1: *S*. *myrtina*, Case 1**).	**Case 1:** On 8/3/2022, an adult female (NOR) consumed these leaves immediately after having severe diarrhea. She then day nested. NOR’s infant did not attempt to eat leaves. NOR’s fecal sample from this day had *Oesophagostomum* (150 EPG). **Case 2:** On 9/26/2021, an adult female (BAH) ate these leaves after day nesting. Her juvenile son, BRI, did not attempt to eat the leaves. BAH’s fecal analysis contained *Oesophagostomum* (1750 EPG), *Ancylostoma (*150 EPG*)*, *Enterobius vermicularis* (100 EPG), and *Trichostrongyloides* (100 EPG).

^1^ Species listed as a putative therapeutic resource for primates in recent review of zoopharmacognosy literature [32]

^2^ Reported in the site’s feeding list for each community

**Fig 1:**
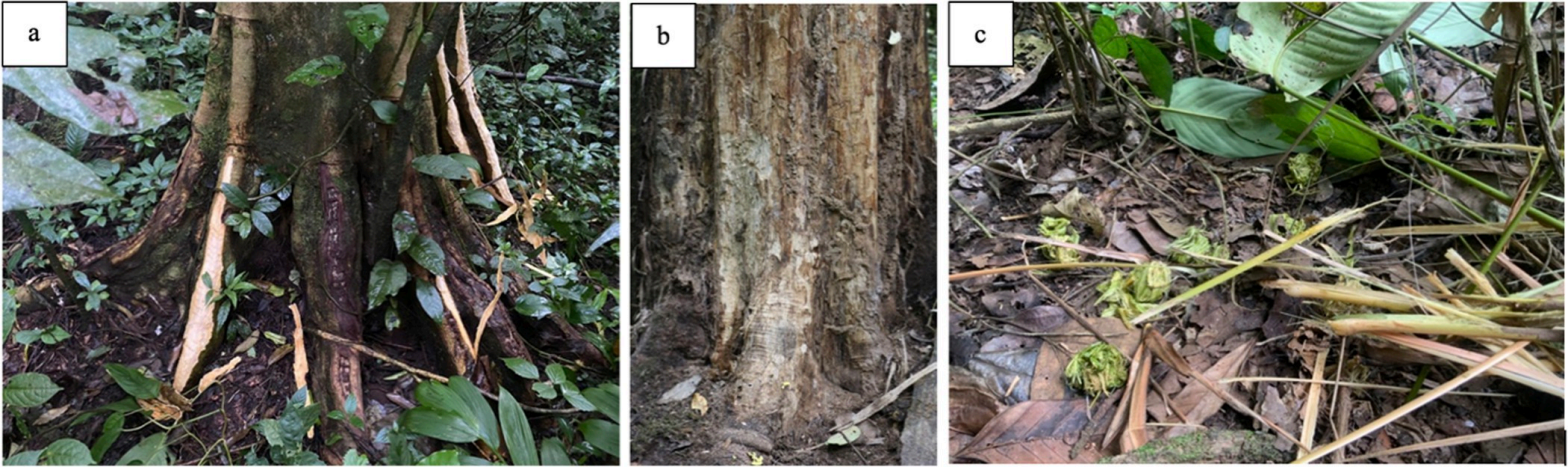
[**a**]: Evidence of F. exasperata bark feeding [**b**] Evidence of C. patens dead wood eating [**c**] Evidence M. leucantha pith-stripping and wadging.

Individuals with injuries were directly observed ingesting *K*. *anthotheca* bark and resin, *W*. *elongata* young leaves, *C*. *alexandri* bark, and *C*. *parasitica* ferns. Individuals exhibiting respiratory symptoms were observed ingesting *C*. *alexandri* bark and *K*. *anthotheca* bark and resin. Individuals with abnormal urinalysis results (e.g., positive for leukocytes, elevated ketones, and presence of blood) were observed feeding on *C*. *patens* dead wood, *K*. *anthotheca* bark and resin, and *M*. *leucantha* pith. Individuals with recent cases of diarrhea were observed consuming *A*. *boonei* and *C*. *patens* dead wood, *K*. *anthotheca* bark and resin, and *W*. *elongata* leaves. Parasitological analyses further suggest individuals with varying degrees of endoparasite infections consumed *S*. *myrtina* and *C*. *alexanderi* bark, *A*. *boonei* and *C*. *patens* dead wood, *K*. *anthotheca* bark and resin, *W*. *elongata* leaves, as well as *A*. *polystachyus* and *M*. *leucantha* pith. On a day when two individuals were observed leaf swallowing, a scientifically established self-medicative behavior, one was observed consuming *K*. *anthotheca* bark and resin, while the other was observed stripping *A*. *polystachyus* pith prior to the event. Ingestion of *F*. *variifolia*, *D*. *dewevrei*, and *S*. *guineense* bark were never directly observed during the study period. Examples of bark feeding, dead wood eating, and pith-stripping marks are shown in **Fig 1**.

### Ethnobotanical review

Based on our analysis of ethnomedicinal literature spanning various African regions from 1976 to 2022, 11 out of the 13 species tested also had documented ethnomedicinal uses (**Table 2**).

**Table 2:** Ethnomedicinal literature review of selected plant species.

Species	Reported Ethnobotanical Uses	Source(s)
*A*. *boonei*	**Bark**: Abortive; Gonorrhea; Asthma; Sores; Ulcers; Pain; Diarrhea; Dysentery; Vermifuge; Liver problems; Dropsy, Inflammation; Edema; Gout; Diabetes; Internal parasites; Dizziness; Breast infection; Nausea, Snakebites; Stomachaches; Malaria; Measles; Uterine fibroid/ovarian cysts; Gynecological lower abdominal and pelvic congestion (PID); Aches from malarial fever; Jaundice **Latex**: Internal parasites; Lactation stimulant	[68, 76, 77]
*A*. *polystachyus*	**Leaf:** Liver/spleen problems; Scabies; Gastroenteritis; Pneumonia; Anthrax; Malaria **Roots:** Gonorrhea; Syphilis; Bleeding; Stabbing pain; Pneumonia	[68, 78–80]
*C*. *alexandri*	**Bark:** Wounds; Acute backache	[81, 82]
*C*. *parasitica*	None known	
*C*. *patens*	**Bark/Sap:** Jaundice; Hepatitis; Stomachache; Tuberculosis; Bronchial affections; Colic; Edemas; Hunchbacks; Rickets; Headache; Pain; Pulmonary troubles; Diarrhea; Hepatitis; Malaria; Measles; Typhoid fever; Menstrual irregularities **Root:** Vermifuge	[77, 83–85]
*D*. *dewevrei*	**Bark:** Pain management; Nasopharyngeal infections; Febrifuges; Venereal diseases; Convulsion; Heartaches; Bodily pains	[77, 83, 86]
*F*. *exasperata*	**General:** Hemorrhoids; Venereal disease; Arthritis; Wounds; Parasites; Diuretic for relaxing uterus; Enhancing uterine contractions **Wood ash/charcoal:** Leprous ulcer; General wounds **Roots:** Asthma; Dyspnea; Venereal disease **Bark:** Intestinal worms; Hemorrhoids; Spleen enlargement; Heart problems; Cough; Dizziness; Facilitation of childbirth; Gonorrhea; Malaria **Bark Sap:** Bleeding; Stimulant; Wounds, Sores, Abscesses; Eye ailments; Stomachache **Leaves**: High blood pressure; Rheumatism; Arthritis; Intestinal pains; Epilepsy; Bleeding; Wounds; Inflammation; Bacterial infections; Fever; Edema; Leprous ulcer; Dermatosis; Abscess; Cough; Cold; Flu; Asthma; Heart disease; Thrush; Gum inflammation; Mouth/ throat ailments; Gastric ulcers; Stomachache; Poison; Kidney disease; Urinary tract infections; Headache; Tumors; Diarrhea; Intestinal parasites **Leaf Shoots**: Dysentery; Jaundice (externally applied); Emetic; Diuretic **Leaf Pulp (external):** Rash; Wounds; Fungal infection; Itching; Ringworm; Rheumatism; Back pain **Dried /Cooked Leaf:** Burns; Gonorrhea	[83, 84, 87]
*F*. *variifolia*	None known	
*K*. *anthotheca*	**Bark:** Colds; Fevers; Pneumonia; Abdominal pain; Vomiting; Gonorrhea; Aphrodisiac; Wounds, Sores, Ulcers; Anemia; Malaria, Bilharzias **Roots:** Anemia; Dysentery; Rectal prolapse	[83, 88, 89]
*M*. *leucantha*	**Roots**: Aphrodisiac **Pith:** Rheumatism; Acne	[68, 90, 91]
*S*. *guineense*	**Bark**: Stomachache; Internal parasites; Purgative; Bodily weakness; Infertility; Abdominal pain; Laxative, Diarrhea; Malaria; Cough; Asthma; Throat problems; Intercoastal pain; Paralysis; Broken bones; Wounds **Roots:** Internal parasites; Purgative; Stomachache; Epilepsy **Leaves:** Enema; Colic; Diarrhea; Abdominal pain; Insanity; Amenorrhea; Cerebral malaria; Intestinal parasites; Stomachache; Insanity; Tonic for pregnancy; Diarrhea; Wounds; Boils; Sprains; Ophthalmia	[68, 83, 92]
*S*. *myrtina*	**Roots**: Intestinal worms; Gonorrhea; Bilharzia; Fever **Leaves:** Ringworm; Wounds; Parturition of placenta and childbirth **Bark/Leaves: Salpingitis**	[68, 93]
*W*. *elongata*	**Leaves:** Bronchitis; Conception aid (women); Stomachache; Food poisoning	[77, 88]

### Production of extracts and sample information

Taxonomic information and extraction details for the 13 plant species studied, including the plant family, local name (when available), plant part used, solvent for extraction, yield of extraction, extract identification numbers (extract IDs), herbarium accession numbers, and collection location are summarized in **Table 3**. Overall, the highest extraction yields were obtained with methanol-water (9/1) as a solvent. The yields from methanol-water extractions for *C*. *parasitica*, *F*. *exasperata* leaves, and *S*. *guineense* stem bark were higher than the other extractions from these samples. The plant samples which had higher yield values with *n*-hexane, such as the leaves of *W*. *elongata* and bark extract of *A*. *boonei*, likely have a higher content of lipids (i.e., fatty molecules).

**Table 3:** Taxonomic information and extraction details of plant samples collected in the Budongo Forest for pharmacological assessment.

Scientific Name (Life form)	Family	Name in Runyoro language & “Common Name”	Plant Part	Extraction Solvent	Extraction Yield [%]	Extract ID	Herbarium Accession Number
***Acanthus polystachyus*** (Terrestrial Herbaceous)	Acanthaceae	n.a.	pith	methanol/water (9:1, *v/v*)	8.4	mwE099	Oxford: 00243136J (E018)
				ethyl acetate	1	eE099	
				n-hexane	0.2	hE099	
***Whitfieldia elongata*** (Climber)	Acanthaceae	n. a.	leaves	methanol/water (9:1, *v/v*)	1.7	mwE090	Oxford: 00243129L (E009)
				ethyl acetate	2.8	eE090	
				n-hexane	2.4	hE090	
***Cleistopholis patens*** (Tree)	Annonaceae	Mubanda murogo “Salt and Oil Tree”	dead wood	methanol/water (9:1, *v/v*)	1.3	mwE091	
				ethyl acetate	0.4	eE091	
				n-hexane	0.1	hE091	
***Alstonia boonei*** (Tree)	Apocynaceae	Mujwa / Kanji	stem bark	methanol/water (9:1, *v/v*)	0.4	mwE092a	Makerere: 51204 (W007)
				ethyl acetate	2.3	eE092a	
				n-hexane	1.6	hE092a	
			dead wood	methanol/water (9:1, *v/v*)	1.2	mwE092b	
				ethyl acetate	0.4	eE092b	
				n-hexane	0.3	hE092b	
***Cynometra alexandri*** (Tree)	Caesalpinioideae	Nyakaimbi Iron Wood	stem bark	methanol/water (9:1, *v/v*)	14	mwE096	Oxford: 00243133G (E015)
				ethyl acetate	2.4	eE096	
				n-hexane	1.1	hE096	
***Khaya anthotheca*** (Tree)	Meliaceae	Munyama White Mahogany	stem bark and resin	methanol/water (9:1, *v/v*)	9.9	mwE088	Oxford: 00243123F (E002)
				ethyl acetate	8.5	eE088	
				n-hexane	6.7	hE088	
***Marantochloa leucantha*** (Terrestrial Herbaceous)	Marantaceae	n. a.	pith	methanol/water (9:1, *v/v*)	0.7	mwE094	Makerere: 51203 (W013)
				ethyl acetate	0.6	eE094	
				n-hexane	0.2	hE094	
***Ficus exasperata*** (Tree)	Moraceae	Musomoro Sandpaper Leaf Tree	stem bark	methanol/water (9:1, *v/v*)	3	mwE093a	Oxford: 00243130D (E012)
				ethyl acetate	0.8	eE093a	
				n-hexane	0.4	hE093a	
			leaves	methanol/water (9:1, *v/v*)	11.5	mwE093b	
				ethyl acetate	3.1	eE093b	
				n-hexane	2.3	hE093b	
***Ficus variifolia*** (Tree)	Moraceae	n.a.	stem bark	methanol/water (9:1, *v/v*)	3.1	mwE097	Makerere: 51195 (W005)
				ethyl acetate	0.5	eE097	
				n-hexane	0.2	hE097	
***Syzygium guineense*** (Tree)	Myrtaceae	n.a.	stem bark	methanol/water (9:1, *v/v*)	9.6	mwE098a	Oxford: 00243135I (E017)
				ethyl acetate	0.8	eE098a	
				n-hexane	0.3	hE098a	
			leaves	methanol/water (9:1, *v/v*)	17	mwE098b	
				ethyl acetate	2.1	eE098b	
				n-hexane	0.5	hE098b	
***Scutia myrtina*** (Scrambling Shrub/Tree)	Rhamnaceace	n. a.	stem bark	methanol/water (9:1, *v/v*)	3.9	mwE089a	Oxford: 00243128K (E007)
				ethyl acetate	0.6	eE089a	
				n-hexane	0.2	hE089a	
			stripped stem bark refuse	methanol/water (9:1, *v/v*)	3.3	mwE089b	
				ethyl acetate	0.6	eE089b	
				n-hexane	0.2	hE089b	
***Christella parasitica*** (Fern)	Thelypteridaceae	n. a.	whole plant	methanol/water (9:1, *v/v*)	14.1	mwE087	Oxford: 00243122E (E001)
				ethyl acetate	2.7	eE087	
				n-hexane	1.7	hE087	
***Desplatsia dewevrei*** (Tree)	Tiliaceae	Omukoma-nyakabita	stem bark	methanol/water (9:1, *v/v*)	3	mwE095	Oxford: 00243132F (E014)
				ethyl acetate	1	eE095	
				n-hexane	0.6	hE095	

### Library screening against multidrug-resistant human and food bacterial pathogens

Initial screening of extracts involved checking for growth inhibition against each bacterium at a concentration of 256 μg/mL. In total, 45 of the 51 plant extracts (88%) showed activity ≥40% inhibition against at least one of the 11 strains and were thus considered active and brought to dose-response experiments to determine their IC_50_ value and MIC. Results from the library screening are reported in **S1 Table in S2 File**. As all tested plant species in the library screen had at least one extract that was active (*in vitro*) against at least one bacterial strain, no entire species was eliminated for further experimentation. However, as no extracts (at any concentration) inhibited the growth of *K*. *pneumoniae*, no further tests were conducted on this bacterium. The extract active against the most bacterial strains (n = 11) was the methanol-water extract of *S*. *guineense* stem bark (mwE098a, active against eight strains), followed by the methanol-water *S*. *guineense* leaves (mwE098b), the ethyl acetate *P*. *patens* dead wood, and the *n*-hexane *A*. *boonei* dead wood (hE092b) extracts, which were each active against seven, seven, and six strains, respectively. The only extract that demonstrated significant inhibition against *P*. *aeruginosa* at the highest test concentration was the methanol-water extract from *S*. *guineense* bark (mwE098a). This was also the only extract to display significant inhibition at 256 μg/mL against *E*. *cloacae*. Of all bacteria in this study, the two strains of *E*. *coli* (DSM 498 and DSM 15076) were the most susceptible, with at least one extract from all plant species inhibiting their growth. The *E*. *coli* strain with nine known antibiotic resistances (DSM 15076) surprisingly showed growth inhibition in 80% of tested extracts.

### Dose-response antibacterial experiments

In dose-response assays, 41 out of the 45 tested extracts (91%) showed activity at ≤256μg/mL, though not all extracts reached MIC values (see **Table 4**). The results, along with standard deviations, are reported in **S2 Table in S2 File**, while **S3 Table in S2 File** provides a summary of the number of strains each extract was active against. The strongest *in vitro* growth inhibition was reported for the methanol-water extract of *K*. *anthotheca* bark and resin (mwE088) against Gram-positive *E*. *faecium* and the *n-*hexane extract of *A*. *boonei* dead wood (hE092b) against Gram-positive *S*. *aureus* (DSM 1104). Both extracts had low IC_50_ values of 16 μg/mL (showing strong inhibition), with MIC values of 32 μg/mL against respective strains. *E*. *faecium* showed the most general susceptibility to *K*. *anthotheca*, with all extracts of this species achieving MIC values (mwE088: 32 μg/mL, eE088: 64 μg/mL, hE088: 128 μg/mL). The ethyl acetate extract of *A*. *boonei* dead wood (eE092b) also strongly inhibited the growth of *E*. *faecium* (IC_50_: 16 μg/mL; MIC: 64 μg/mL), as did the *n-*hexane extract of *A*. *boonei* dead wood, producing an IC_50_ value of 16 μg/mL but failing to reach a MIC value. *S*. *aureus* (DSM 1104) was also highly susceptible to the ethyl acetate extracts of *A*. *boonei* dead wood (IC_50_: 32 μg/mL; MIC: 128 μg/mL).

**Table 4:** IC50 and MIC values obtained from in vitro dose-response study on bacterial growth inhibition.

Scientific name	Extract ID	*S*. *aureus* DSM 1104		*S*. *aureus* DSM 18827		*A*. *baumannii* DSM 102929		*E*. *cloacae* DSM 30054		*P*. *aeruginosa* DSM 1117		*E*. *faecium* DSM 13590		*E*. *coli* DSM 498		*E*. *coli* DSM 1576		*S*. *maltophilia DSM 50170*		*S*. *enterica subsp*. *enterica DSM 11320*	
		IC_50_	MIC	IC_50_	MIC	IC_50_	MIC	IC_50_	MIC	IC_50_	MIC	IC_50_	MIC	IC_50_	MIC	IC_50_	MIC	IC_50_	MIC	IC_50_	MIC
*C*. *parasitica*	eE087	-	-	-	-	-	-	-	-	-	-	-	-	>256	>256	-	-	-	**-**	-	-
	hE087	-	-	-	-	-	-	-	-	-	-	-	-	>256	>256	**128**	>256	-	**-**	-	-
*K*. *anthotheca*	mwE088	-	-	-	-	>256	>256	-	-	-	-	**16**	32	>256	>256	**16**	256	**64**	>256	-	-
	eE088	-	-	-	-	**-**	-	-	-	-	-	**64**	64	>256	>256	**64**	>256	-	-	-	-
	hE088	-	-	-	-	**-**	-	-	-	-	-	**64**	128	-	-	**64**	>256	-	-	**256**	>256
*S*. *myrtina*	mwE089a	-	-	-	-	**-**	-	-	-	-	-	-	-	>256	>256	**128**	>256	-	-	-	-
	eE089a	-	-	-	-	**-**	-	-	-	-	-	**64**	>256	>256	>256	**256**	>256	-	-	-	-
	hE089a	-	-	-	-	**-**	-	-	-	-	-	-	-	>256	>256	-	-	-	-	-	-
	mwE089b	-	-	-	-	**-**	-	-	-	-	-	-	-	-	-	**256**	>256	-	-	-	-
	eE089b	-	-	-	-	>256	>256	-	-	-	-	**128**	>256	>256	>256	**256**	>256	-	-	-	-
	hE089b	-	-	>256	>256	**256**	>256	-	-	-	-	-	-	-	-	**256**	>256	-	-	-	-
*W*. *elongata*	mwE090	-	-	**-**	-	-	-	-	-	-	-	-	-	-	-	**128**	>256	-	-	-	-
	eE090	-	-	**-**	-	-	-	-	-	-	-	**64**	128	>256	>256	**256**	>256	-	-	-	-
*C*. *patens*	mwE091	-	-	**-**	-	-	-	-	-	-	-	-	-	-	-	**128**	>256	-	-	-	-
	eE091	**128**	256	**-**	-	>256	>256	-	-	-	-	**64**	64	>256	>256	**128**	>256	**256**	>256	>256	>256
	hE091	-	-	**-**	-			-	-	-	-	**64**	>256	-	-	**128**	>256	-	-	-	-
*A*. *boonei*	mwE092a	-	-	**-**	-	-	-	**-**	**-**	-	-	-	-	-	-	**128**	>256	-	-	-	-
	hE092a	-	-	**-**	-	>256	>256	-	-	-	-	-	-	-	-	-	-	-	-	-	-
	mwE092b	-	-	**-**	-	-	-	-	-	-	-	-	-	**256**	>256	**32**	>256	-	-	-	-
	eE092b	**32**	128	-	-	>256	>256	-	-	-	-	**16**	64	>256	>256	**128**	>256	-	-	-	-
	hE092b	**16**	32	**32**	>256	-	-	-	-	-	-	**16**	>256	>256	>256	**256**	>256	-	-	**256**	256
*F*. *exasperata*	mwE093a	-	-	-	-	-	-	-	-	-	-	-	-	-	-	**128**	>256	-	-	-	-
	eE093a	-	-	-	-	-	-	-	-	-	-	**32**	128	>256	>256	**128**	>256	-	-	-	-
	hE093a	-	-	-	-	-	-	-	-	-	-	**32**	>256	-	-	-	-	-	-	-	-
	mwE093b	-	-	-	-	-	-	-	-	-	-	-	-			**256**	>256	-	-	-	-
	hE093b	-	-	-	-	-	-	-	-	-	-	-	-	>256	>256	**256**	>256	-	-	-	-
*M*. *leucantha*	mwE094	-	-	-	-	-	-	-	-	-	-	-	-	-	-	**128**	>256	-	-	-	-
	eE094	-	-	-	-	>256	>256	-	-	-	-	-	-	>256	>256	**256**	>256	-	-	-	-
	hE094	-	-	-	-	-	-	-	-	-	-	-	-	-	-	**256**	>256	-	-	-	-
*D*. *dewevrei*	mwE095	-	-	-	-	>256	>256	-	-	-	-	-	-	>256	>256	**256**	>256	**256**	>256	-	-
	eE095	-	-	-	-	-	-	-	-	-	-	-	-	>256	>256	**256**	>256	-	-	-	-
	hE095	-	-	-	-	-	-	-	-	-	-	-	-	-	-	>256	>256	-	-	-	-
*C*. *alexandri*	mwE096	>256	>256	-	-	>256	>256	-	-	-	-	-	-			**256**	>256	**256**	>256	-	-
	eE096	-	-	-	-	-	-	-	-	-	-	-	-	>256	>256	**256**	>256	-	-	-	-
	hE096	-	-	-	-	-	-	-	-	-	-	-	-	>256	>256	**256**	>256	-	-	-	-
*F*. *variifolia*	eE097	-	-	-	-	-	-	-	-	-	-	**64**	>256	>256	>256	**256**	>256	-	-	-	-
	hE097	-	-	-	-	-	-	-	-	-	-	-	-	>256	>256	**256**	>256	-	-	-	-
*S*. *guineense*	mwE098a	>256	>256	>256	>256	**64**	>256	**128**	>256	**64**	>256	-	-	>256	>256	**64**	>256	**32**	256	-	-
	eE098a	-	-	>256	>256	**128**	>256	256	>256	**-**	**-**	-	-	>256	>256	**128**	>256	**64**	256	-	-
	hE098a	-	-	-	-	-	-	-	-	**-**	**-**	-	-	-	-	**128**	>256	-	-	-	-
	mwE098b	**128**	>256	>256	>256	**128**	>256	**128**	>256	**-**	**-**	-	-	**128**	>256	**32**	128	**32**	>256	-	-
	eE098b	-	-	>256	>256	>256	>256	**256**	>256	**-**	**-**	-	-	>256	>256	**128**	256	**128**	>256	-	-
	hE098b	-	-	>256	>256	>256	>256	>256	>256	**-**	**-**	-	-			**256**	>256	**128**	>256	-	-
*A*. *polystachius*	eE099	-	-	-	-	-	-	-	-	-	-	**128**	256	>256	>256	**256**	256	-	-	-	-
	hE099	**256**	>256	-	-	-	-	-	-	-	-	**32**	128	>256	>256	**256**	>256	-	-	-	-
**Vancomycin**		<1	2	<1	1	>64	>64	>64	>64	>64	>64	>64	>64	n.t.		n.t.		n.t.		n.t	
**Gentamicin**		0.125	0.25	0.5	1	>64	>64	n.t.		n.t.		8	>64	<1	1	n.t.		8	8	n.t.	
**Ciprofloxacin**		n.t.		n.t.		>64	>64	n.t.		n.t.		n.t.		n.t.		n.t.		n.t.		n.t.	
**Tetracyclin**		n.t.		n.t.		1	2	n.t.		n.t.		n.t.		<1	2	n.t.		n.t.		1	2
**Chloramphenicol**		2	8	4	16	32	64	2	4	32	>64	-	4	n.t.		<1	4	n.t.		4	2

**-** = not taken to dose-response (<40% growth inhibition at 256 μg/mL in the library screen).

**n.t.** = not tested.

**NB:** Only extracts showing growth inhibition ≥40% in the library screen at 256 μg/mL are listed. IC_50_ and MIC values are expressed as concentration (μg/mL). The concentrations tested ranged from 256μg/mL to 4 μg/mL.

**NB:** IC_50_ values ≤256 μg/mL are in **bold.**

Only one extract, the methanol-water extract of *S*. *guineense* bark (mwE098a), was active against the gram-negative *P*. *aeruginosa*. This extract exhibited moderate growth inhibition (IC_50_: 64 μg/mL) with no MIC value reached. Despite *E*. *coli* (DSM 498) being highly susceptible on the library screen, only two extracts, the methanol-water extract of *A*. *boonei* dead wood (mwE092b; IC_50_: 256 μg/mL) and the methanol-water extract of *S*. *guineense* leaves (mwE098b; IC_50_: 128 μg/mL), reached IC_50_ values at the concentration range tested, with no MICs reached. Interestingly, the strain of *E*. *coli* with nine known resistances (DSM 1576) was more susceptible, with 89% (N = 40) of extracts achieving IC_50_ values ≤ 256 μg/mL. The most active extract against this strain was the methanol-water extract of *K*. *anthotheca* (mwE088; IC_50_: 16 μg/mL; MIC: 256 μg/mL). *S*. *guineense* exhibited the highest overall inhibition of *S*. *maltophilia*, with all extracts except hE098a displaying IC_50_ values of ≤ 256 μg/mL against the bacterium. At the concentration range tested, no extracts yielded MIC values for *S*. *aureus* (DSM 18827), *A*. *baumannii*, *E*. *cloacae*, *P*. *aeruginosa* or *E*. *coli* (DSM 498).

### Anti-inflammatory COX-2 inhibition library screen

Results from the *in vitro* COX-2 inhibition library screen at descending concentrations are reported in **S4 Table in S2 File**. At the initial concentration of 50 μg/mL, 43 out of 51 extracts (84%) exhibited an enzyme inhibition of at least 50%, displaying anti-inflammatory activity. This included at least one extract of every plant species. In the next stage of screening, at 10 μg/mL, 18 samples were eliminated. During the final step, at 5 μg/mL, five more were eliminated. The remaining 17 extracts from 10 plant species which displayed inhibition ≥50% at 5 μg/mL, were then introduced to dose-response experiments. The ethyl acetate *S*. *myrtina* bark extract (eE089b) was taken to the COX-2 dose-response despite not showing inhibition past 50 μg/mL, as it almost reached the selection limit during analysis and had a relatively high standard deviation. No extracts from *W*. *elongata*, *C*. *patens* or *D*. *dewevrei* showed COX-2 inhibition at 5 μg/mL and thus were excluded from further testing.

### COX-2 inhibition dose-response experiments

The most active COX-2 inhibitors were extracts from *K*. *anthotheca* (mwE088; hE088; eE088), *C*. *parasitica* (mwE087; hE087), *F*. *exasperata* (hE093a; eE093a), *S*. *myrtina* (hE089a; eE089b), *F*. *variifolia* (eE097; hE097), *A*. *polystachyus* (hE099; eE099), *M*. *leucantha* (hE094), *S*. *guineense* (hE098a), *A*. *boonei* (hE092b), and *C*. *alexandri* (hE096). Results are reported in **Table 5**. The strongest COX-2 inhibitor was the *K*. *anthotheca* methanol-water bark and resin extract (mwE088) (IC_50_ of 0.55 μg/mL), followed by the *C*. *parasitica* methanol-water fern extract (mwE087) (IC_50_ of 0.81 μg/mL). In contrast, all extracts of the species *W*. *elongata*, *C*. *patens*, and *D*. *dewevrei* failed to show ≥50% inhibition, mostly at the second screening concentration (10 μg/mL). *W*. *elongata* extracts notably showed low activity in both antibacterial and COX-2 inhibition assays.

**Table 5:** Results of in vitro COX-2 inhibition studies.

				IC_50_ ± SEM
Extract ID	Plant Species	Plant Part	Type of Extract	COX-2
**mwE088**	*K*. *anthotheca*	stem bark & resin	methanol/water (9:1, *v/v*)	0.55 ± 0.14
**mwE087**	*C*. *parasitica*	fern	methanol/water (9:1, *v/v*)	0.81 ± 0.11
**hE088**	*K*. *anthotheca*	stem bark &resin	*n*-hexane	1.02 ± 0.01
**hE093a**	*F*. *exasperata*	stem bark	*n*-hexane	1.06 ± 0.09
**hE089a**	*S*. *myrtina*	stem bark	*n-*hexane	1.19 ± 0.05
**eE097**	*F*. *variifolia*	stem bark	ethyl acetate	1.20 ± 0.27
**eE088**	*K*. *anthotheca*	stem bark &resin	ethyl acetate	1.30 ± 0.06
**hE099**	*A*. *polystachyus*	pith	*n*-hexane	1.63 ± 0.98
**hE094**	*M*. *leucantha*	pith	*n*-hexane	1.79 ± 0.14
**eE093a**	*F*. *exasperata*	stem bark	ethyl acetate	2.11 ± 0.08
**hE098a**	*S*. *guineense*	stem bark	*n*-hexane	2.42 ± 0.28
**hE092b**	*A*. *boonei*	dead wood	*n*-hexane	2.74 ± 0.37
**hE087**	*C*. *parasitica*	fern	*n*-hexane	3.18 ± 0.99
**hE097**	*F*. *variifolia*	stem bark	*n*-hexane	3.37 ± 0.59
**hE096**	*C*. *alexandri*	stem bark	*n-*hexane	4.83 ± 0.52
**eE089b**	*S*. *myrtina*	stem bark (refuse)	ethyl acetate	7.49 ± 0.52
**eE099**	*A*. *polystachyus*	pith	ethyl acetate	7.83 ± 0.56
**positive control**	*DuP-769*		(pure compound)	0.93 ± 0.20

NB: Extracts are sorted from highest to lowest COX-2 sensitivity; IC_50_ values are provided in μg/mL (positive control: ng/mL)

SEM = standard error of the mean

## Discussion

### Plant species with strong pharmacological activity

This study provides the first pharmacological and behavioral evidence of its kind, based on *in situ* sampling, for the medicinal benefits of bark feeding, dead wood eating, and non-bitter pith stripping behaviors in Budongo chimpanzees. In the following sub-sections, we describe and discuss specific results from five of the tested plant species in further detail. For scope, we selected the two species with the strongest antibacterial properties (*K*. *anthotheca* and *A*. *boonei*) to profile, both of which were the only species to reach 40% inhibition at 16 μg/mL. We also selected *C*. *parasitica to* discuss as this species, along with *K*. *anthotheca*, exhibited the strongest anti-inflammatory properties. We then discuss results from our *S*. *guineense* samples, as this species was effective against the most bacterial strains in our antibacterial assays. Lastly, we selected *S*. *myrtina*, as we have behavioral evidence and health data that anecdotally support the use of this species for therapeutic self-medication by Budongo chimpanzees.

***Alstonia boonei*.** Numerous *in vitro* and *in vivo* studies, reviewed by Adotey [76], have reported pharmacological activity in *A*. *boonei* bark. However, none of these studies investigated dead wood samples of *A*. *boonei*. Consistent with these findings, we found high levels of antibacterial and anti-inflammatory activity in the extracts of this species. Interestingly, extracts from *A*. *boonei* dead wood generally exhibited higher activity than living bark. This difference could be due either to a change in active ingredient composition, or possible fungal growth following the tree’s death. While the *A*. *boonei* dead wood *n*-hexane extract (hE092b) exhibited strong growth inhibition against *S*. *aureus* (DSM 1104; DSM 18827) and *E*. *faecium* at low concentrations in the dose-response assays, the *n*-hexane bark extract (hE092a) showed no activity <256 μg/mL. Similarly, the ethyl acetate extract of dead wood (eE092b) also strongly inhibited *S*. *aureus* (DSM 1104) (IC_50_: 16 μg/mL; MIC: 128 μg/mL) and *E*. *faecium* (IC_50_: 16 μg/mL; MIC: 64 μg/mL), while the ethyl acetate bark extract of this species did not even exhibit enough inhibition in the antibacterial library screen to be taken to dose-response assays. However, the methanol-water extract of *A*. *boonei* bark (mwE092a) did show activity against *E*. *coli* (DSM 498) (IC_50_: 128 μg/mL), as did the methanol-water dead wood extract (mwE092a) (IC_50_: 128 μg/mL), with no MIC values reached in either case. Overall, extracts from *A*. *boonei* displayed more potent activity in Gram-positive bacteria, although this effect is more apparent in dead wood than stem bark. In the COX-2 inhibition assays, the *n*-hexane extract of *A*. *boonei* dead wood also showed strong anti-inflammatory inhibition, while the *n*-hexane extract of the bark only exhibited weak inhibition (at the highest test concentration of 50 μg/mL).

*A*. *boonei* is a known medicinal plant across East Africa, commonly used for a variety of reproductive, bacterial, and gastro-intestinal issues, as well as for snake bites, asthma, and dizziness [68, 76, 77]. The bark and latex are intensely bitter, a reliable signal of the presence of bioactive secondary compounds and toxicity [94–96]. Budongo chimpanzees in both communities have been reported to consume both bark and dead wood of *A*. *boonei*, often travelling long distances to access these trees and only consuming small amounts of bark per feeding bout [45]. In an observation reported in this study (see **Table 1: *A*. *boonei*, Case 1**), three males ingested *A*. *boonei* dead wood while outside the community’s core area for 1-minute. Two days before the event, one of the individuals had been observed with diarrhea, while also shedding visible tapeworm proglottids (*Bertiella* sp.). This sample also contained unidentified protozoa, and *Taenia* sp. eggs. Pebsworth et al. [34] also reported an event in which four adult males, all with diverse parasite loads, traveled to a large *A*. *boonei* tree and ingested bark.

In the long-term site data, *A*. *boonei* bark ingestion was only documented 17 times between 2008–2021 [45], although this behavior was not systematically reported. In addition, the direct observation of only one *A*. *boonei* dead wood eating event, and no *A*. *boonei* bark ingesting events over the two four-month periods of observation in this study, suggest that consumption of this species is relatively rare across both communities. While specific pathogenic catalysts for selection of this species remain unknown, based on pharmacological, ethnobotanical, and behavioral data, we propose that *A*. *boonei* may be a therapeutic self-medicative resource for Budongo chimpanzees. The relatively strong inhibitory activity of this species against *S*. *aureus*, a bacteria associated with causing contamination on the skin leading to chronic wounds [97], as well as its anti-inflammatory properties, suggests that *A*. *boonei* ingestion may have beneficial effects in wound care contexts.

***Khaya anthotheca*.** Previous studies have demonstrated that *K*. *anthotheca* bark contains biologically active compounds like gedunins, mexicanolide, phragmalin, and andirobins [98]. One limonoid identified in the species, anthothecol, has anti-cancer properties [99]. A study by Obbo et al. [100] on *K*. *anthotheca* bark collected in the Budongo Forest, found strong antiprotozoal activity against *Plasmodium falciparum* (IC_50_ 0.96 μg/mL) and *Trypanosoma brucei rhodesiense* (IC_50_ 5.72 μg/mL). A related species, *K*. *senegalensis*, has been shown to cause cell lysis in some gram-negative bacteria, including *Salmonella Typhimurium*, *Escherichia coli*, *Shigella* sp. and *Salmonella* sp., by targeting cytoplasmic membranes [101].

In our antibacterial library screen, of all extracts tested, only the methanol-water extract inhibited growth of *A*. *baumannii* (although no IC_50_ values were reached in dose-response). The methanol-water extract also inhibited the growth of *E*. *coli* (DSM 498) in the library screen, as did the ethyl acetate (eE088) extract, though again no IC_50_ values were reached. In our antibacterial dose-response assays, all extracts of *K*. *anthotheca* stem bark and resin exhibited strong inhibition against the Gram-positive *E*. *faecium*. The most active extract against this strain, which was also the strongest antibacterial result reported in this study, was methanol-water (mwE088) (IC_50_: 16 μg/mL; MIC: 32 μg/mL). All extracts of this species were also found to inhibit *E*. *coli* (DSM 1576) in the dose-response experiments, with the methanol-water extract once again also showing the strongest inhibition (IC_50_: 16 μg/mL; MIC: 256 μg/mL). This extract also inhibited the growth of *S*. *maltophilia* (IC_50_: 64 μg/mL) in the library screen. Only weak inhibition was found against the food pathogen *S*. *enterica* (*n*-hexane extract, IC_50_: 256 μg/mL).

*K*. *anthotheca* exhibited potent anti-inflammatory activity. Of all extracts tested, the methanol-water *K*. *anthotheca* extract (mwE088) displayed the strongest COX-2 inhibition activity (IC_50_: 0.55 μg/mL). Past phytochemical studies on methanol and ethanol-water stem bark extracts from the related species, *K*. *senegalensis*, revealed many phenolic compounds, including flavonoids and tannins e.g., [101, 102]. Flavonoids act on the inflammatory response, and may block molecules like COXs, cytokines, nuclear factor-кB and matrix metalloproteinases [103]. Some tannins have also been proven to have strong free radical-scavenging and antioxidant activities [104]. These compounds are antagonists of particular hormone receptors or inhibitors of particular enzymes such as COX enzymes [103]. If *Khaya* species are phytochemically similar, this could help explain *K*. *anthotheca*’s strong COX-2 inhibitory activity.

Across Africa, *K*. *anthotheca* is traditionally used for ailments including allergies, fever, headaches, jaundice, bacterial infections, and as a disinfectant for bleeding wounds [105–107]. Our behavioral observations suggest that this species is also a common resource for Sonso chimpanzees, with a total of 65 feeding events recorded throughout the first field season. Of these events, several involved individuals with imbalanced health states (see **Table 1: *K*. *anthotheca*)**. On at least three independent occasions, *K*. *anthotheca* bark and resin were consumed by wounded individuals. Two adult females on different days tested positive for leukocytes on urinalysis tests within hours of ingesting *K*. *anthotheca*, suggesting the presence of infection. One of these individuals was also experiencing severe diarrhea the day prior, the other was found to have trace levels of blood in her urine. A juvenile female with a persistent cough was also observed consuming *K*. *anthotheca* bark. On several occasions individuals with high parasite loads or diverse species infection were observed targeting this resource while shedding tapeworm proglottids (*Bertiella* sp.). An elderly female was also observed eating bark and resin a few hours prior to leaf-swallowing, a well-established self-medicative behavior known to rid the gut of endoparasites [9, 23]. The frequency of *K*. *anthotheca* ingestion in the Sonso diet during this period, suggests that individuals have consistent exposure to the antibacterial and anti-inflammatory compounds present in this species. Whether this is a case of passive prevention through intake of a medicinal food, or therapeutic self-medication for a common and wide-spread condition will need further investigation. If used therapeutically, our results suggest this species could be used for treating wounds, bacterial or infections, and/or reducing internal parasite loads.

#### Christella parasitica

Extracts of *C*. *parasitica* produced notably high anti-inflammatory activity in COX-2 testing, with the methanol-water extract (mwE087) achieving an IC_50_ value of 0.81 μg/mL. This same extract, however, exhibited the lowest general activity in the antibacterial library screen. The only antibacterial activity from this species was on *E*. *coli* (DSM 498) by the ethyl acetate and *n-*hexane extracts (eE087; hE087), and on *E*. *coli* (DSM 1576) by the n-hexane extract (hE087). The *n*-hexane extract reached an IC_50_ of 128 μg/mL in dose-response assays with no MIC value. Prior to this study, there had been limited pharmacological testing on *C*. *parasitica* (though see [108]), so comparison across studies is not possible.

When we considered the associated behavioral observation involving *C*. *parasitica*, we found a notable relevance to our pharmacological results (see **Table 1: *C*. *parasitica*, Case 1**). This observation involved a wounded Sonso adult male (PS) travelling outside of his core area with a large group. It was unclear if this was an inter-community patrol. PS had been observed earlier in the day with a severe hand injury which impacted his mobility, though no open wound was observed. PS separated himself from the group and moved a few meters to a patch of ferns where he began consuming the leaflets. The bout lasted approximately 3-minutes. No other group members were observed feeding on this species, and this was only the second case of fern ingestion reported in Budongo in over 30-years of observations (unpublished site data). Health states of individuals from the past event were unfortunately not recorded. Whether or not *C*. *parasitica*’s highly anti-inflammatory properties were the principal motivator for the selection of this species remains unknown, however, regardless of intention, this plant may have benefitted PS by reducing pain and swelling in his injured hand.

#### Syzygium guineense

*S*. *guineense* bark and leaves have both previously been found to exhibit a range of pharmacological activity, reviewed by Uddin et al. [109]. The antioxidant, analgesic, and anti-inflammatory activities of this plant have been attributed to flavonoids, tannins, saponins, carbohydrates, alkaloids, and cardiac glycosides in the extracts [109–112]. In our assays, *S*. *guineense* bark exhibited high antibacterial growth inhibition effects *in vitro*. The methanol-water bark extract (mwE098a) showed some level of inhibition against all bacteria tested in the dose-response assays, except for *E*. *faecium* and *S*. *enterica*. This was also the only extract, out of all tested, to inhibit growth of *P*. *aeruginosa* (IC_50_: 64 μg/mL; MIC: >256 μg/mL) a pathogen known to cause infections in the blood, lungs, and other body parts after surgeries [113], and was one of two extracts to reach a MIC value against *S*. *maltophilia* (IC_50_: 32μg/mL; MIC: 256 μg/mL). The other extract to reach a MIC value was the ethyl acetate *S*. *guineense* bark extract (eE098a; IC_50_: 64 μg/mL; MIC: 256 μg/mL). All bark and leaf extracts showed strong inhibition against *E*. *coli* (DSM 1576) in the dose-response assays, with the strongest results coming from the methanol-water extracts (mwE098a and mwE098b). All bark and leaf extracts of this species, except for the *n*-hexane bark extract (hE098a), inhibited *E*. *cloacae*, and were the only extracts in the study to do so. *E*. *cloacae*, while part of normal intestinal flora, can cause UTI’s and respiratory infections in humans [114]. *S*. *guineense* extracts were also the only extracts to inhibit *A*. *baumannii* at a concentration <256 μg/mL, with the methanol-water bark extract showing the strongest inhibition. *A*. *baumannii* can cause infections in wounds, blood, urinary tracts, and lungs [115]. The efficacy of methanolic extracts from this species suggests that the active compounds are polar molecules. In the anti-inflammatory COX-2 inhibition dose-response assays, only the *n*-hexane bark extract displayed strong inhibitory effects (IC_50_: 2.42 μg/mL), while the other extracts failed to exhibit significant activity during the pre-screening or ≥ 50% inhibition at 10 μg/mL. The COX-2 inhibition assays showed no inflammatory inhibition amongst leaf extracts at tested concentrations.

*S*. *guineense* can be found throughout Sub-Saharan Africa and is a common traditional medicine, for malaria [116]. The bark is also used for stomach aches, diarrhea, internal parasites, and infertility [68, 109]. Ingestion of *S*. *guineense* bark is rare in Budongo, with no direct observations in either community throughout the study period, and only six total cases between 2008–2021 documented in the site’s long-term data. No observations of leaf ingestion of this species have ever been reported. The infrequent ingestion of *S*. *guineense* bark implies a more targeted use, making it unlikely to be a medicinal food. Instead, our pharmacological findings make this resource a strong candidate as a putative, therapeutic self-medicative resource. Unfortunately, as there is currently no health data associated with individuals who have recently consumed *S*. *guineense* bark, we do not yet know which properties chimpanzees may be targeting. However, based on pharmacological results, we recommend further investigation into this species as a curative agent for respiratory-related infections.

#### Scutia myrtina

Kritheka et al. [117] in their study on the bioactivity of *S*. *myrtina*, found *in vivo* evidence that this species possesses dose-dependent anti-inflammatory, antimicrobial, and antifungal properties. Across our antibacterial assays, the bark sample of this species collected from the stem inhibited *E*. *faecium* (eE089a) and *E*. *coli* DSM 1576 (eE089a; mwE089a) in dose-response tests at concentrations ≤256 μg/mL. The refuse sample, collected from the ground below the plant’s stem, inhibited *A*. *baumannii* (hE089b), *E*. *faecium* (eE089b), and *E*. *coli* DSM 1576 (mwE089b; eE089b; hE089b) in dose-response tests below the specified concentration. Interestingly, the refuse sample inhibited more bacteria species overall than the fresh bark. The most potent antibacterial growth inhibition effects came from the ethyl acetate bark sample against *E*. *faecium* (eE089a; IC_50_: 64 μg/mL), though no MIC value was reached. In the COX-2 inhibition assays, the *n-*hexane bark extract had the fifth strongest inhibitory effect *in vitro* (hE089a; IC_50_: 1.19 μg/mL) out of all samples, while the ethyl acetate refuse bark sample was less potent, though still moderately active (E089b; IC_50_: 7.49 μg/mL).

As far as the authors know, this is the first published report presenting both behavioral and pharmacological evidence for *S*. *myrtina* bark as a putative medicinal resource amongst free-ranging chimpanzees (though see [118] for evidence based on food-combinations). Our behavioral observations indicate that an individual with a diverse and intense parasite infection deliberately sought out the bark of this species. The Budongo chimpanzees may, therefore, utilize *S*. *myrtina* as an anthelminthic. Across traditional accounts from multiple regions, *S*. *myrtina* is commonly used by people as an anthelminthic to treat intestinal worms [68], while aerial parts are also used to treat various bacterial infections. As we were not able to conduct urinalysis on the consumer during or after this event, we cannot determine whether the individual also harbored a bacterial infection at the time of ingestion. However, this possibility cannot be ruled out. Based on these findings, we propose *S*. *myrtina* be added to the list of putative chimpanzee self-medication behaviors as a treatment for internal parasites, and we encourage further exploration into the other specific chimpanzee health conditions that this species may help ameliorate.

### Assessment of putative self-medicative behaviors

We synthesized pharmacological and behavioral evidence to assess therapeutic use of species associated with bark feeding, dead wood eating, and pith stripping behaviors. A summary of the antibacterial and anti-inflammatory results for each species is reported in **S3 Table in S2 File**. Overall, stem bark and dead wood samples were notable for their activity. Bark samples from every species showed >40% antibacterial inhibition against at least one bacterial strain. This activity was also true of the dead wood samples. When plant parts of the same species were tested (*S*. *guineense* and *F*. *exasperata*), barks generally exhibited more potent antibacterial and COX-2 inhibition activity than the leaves, likely to do with the higher concentration of plant secondary metabolites in bark. Our findings offer strong support that bark and dead wood eating of *certain* species could constitute novel self-medicative behaviors in wild chimpanzees. We also encourage more investigation into the bioactivity of non-bitter pith stripping, as the pith of *A*. *polystachius* showed strong antibacterial activity against *E*. *faecium* (hE099; IC_50_: 32 μg/mL; MIC: 128 μg/mL), and the piths of both *A*. *polystachius* and *M*. *leucantha* demonstrated significant anti-inflammatory properties at low concentrations. Future primatological research should prioritize the establishment of multi-disciplinary long-term projects that look systematically at health states of individuals who engage in bark, dead wood, and pith ingestion behaviors. We also encourage further pharmacological testing on other species used for these behaviors in Budongo and across primate field sites.

### Drug discovery

Multidisciplinary studies on this topic have potential to lead to the discovery of new medicines which may benefit our own species [119–122]. Historically, PSMs have played a major role in the development of modern human medicine, and even today, a large portion of medicines are derived either directly or indirectly from plants and other natural materials [123–127]. Antimicrobial resistance is rising to dangerously high levels according to the World Health Organization [128] requiring the rapid creation of new antibacterial treatments. Infections caused by multi-drug resistant bacteria kill hundreds of thousands of people annually. Our findings of strong antibacterial growth inhibition across numerous plant species growing in Budongo have promising implications for our ability to discover novel compounds in existing forest habitats. Extracts should also be tested against additional bacteria and for anti-virulence effects, e.g., inhibition and disruption of biofilm formation, quorum sensing and toxin production, pursuing development of new therapeutic strategies that apply less evolutionary pressure, likely resulting in emergence of less antibiotic resistances in the future. Phytochemical characterization using advanced techniques, such as LC-ToF-MS and NMR, as well as potentially AI-assisted untargeted metabolomics approaches, are now needed to identify substances present in the most active extracts. This may eventually lead to the isolation and structure elucidation of yet unknown active ingredients and make way for determining their pharmacological selectivity and toxicity, while also taking potential synergistic effects into account.

Simultaneously, we are currently faced with a pressing need for more effective treatments to combat symptoms of acute inflammation and mediate long-term consequences of chronic inflammatory diseases [129]. The prostaglandin-producing cyclooxygenase-2 (COX-2) mediates and regulates pain, fever, wound inflammation, and many other medical disorders, as it plays a crucial role in the host organism’s defense against pathogens and injury. COX-2 inhibition has the same mechanism of action as non-steroidal anti-inflammatory drugs (NSAIDs). While inflammation is a normal part of the body’s defense against injury or infection, it can be damaging when occurring in healthy tissues or over a protracted period. Chronic inflammation can lead to cardiovascular diseases (CVD) and cancer, the two leading global causes of death [130]. Past studies have shown that the IC_50_ values of Aspirin and ibuprofen (pure compounds and common NSAIDs) are 210 μg/mL and 46 μg/mL respectively for COX-2, and 5 μg/mL and 1 μg/mL respectively for COX-1 [131, 132]. The *in vitro* COX-2/COX-1 selectivity ratio for Aspirin and ibuprofen is 42 and 46 respectively. Surprisingly, the 17 most active extracts in our COX-2 assays display lower IC_50_ values than these popular NSAIDs, meaning our extracts have more potent inhibitory effects on the inhibition of COX-2 than the most common anti-fever and anti-pain drugs on the market. While COX-1 assays were beyond the scope of this study, future research should investigate COX-1 inhibition activity of these 17 extracts to calculate COX-2/COX-1 selectivity ratios. Doing so will allow for preliminary assessment of potential side effects, selectivity, and efficacy before future *in vivo* experiments can commence.

### Future directions

Future research on this topic would benefit from the inclusion of control samples (plants or plant parts not consumed by chimpanzees); however, in this study, assay costs were a prohibiting factor. Additional information regarding the nutritional and mineral content of the species mentioned in this study is needed to better understand the motivations for ingestion. However, bioactivity and nutritional/mineral content are by no means mutually exclusive. It is, therefore, highly likely that these resources provide multiple benefits to consumers.

Future studies should also consider ecological variables. For example, different individual plants of the same species should be tested across habitat types to determine whether bioactivity varies based on location, age, life history, or time of harvest. Situating samples in their ecological context will provide a better understanding of whether chimpanzees select resources based on species alone, or other more nuanced criteria. Lastly, climatic studies in combination with pharmacological testing should examine how climate change may impact bioactivity of these plants, as shifting weather patterns have already been shown to alter nutritional content [133]. This information will be critical for establishing protected habitats that can sustain healthy, wild, primate populations.

## Conclusions

As we learn more about the pharmacological properties of plants ingested by chimpanzees in the wild, we can expand our understanding of their health maintenance strategies. Our results provide pharmacological evidence, from *in vitro* assays of plant parts consumed by wild chimpanzees collected *in situ*, for the presence of potent bioactive secondary plant metabolites in Budongo chimpanzee diets for a variety of potential illnesses previously not considered. Whether these resources are consumed intentionally as a form of therapeutic self-medication or passively as medicinal foods, must be assessed on a case-by-case basis, taking behavioral observations into account.

For the field of zoopharmacognosy to progress, we encourage continued multidisciplinary collaboration between primatologists, ethnopharmacologists, parasitologists, ecologists, and botanists [9]. Beyond improving our broad understanding of chimpanzee health maintenance, multidisciplinary studies will benefit our own species, potentially leading to the discovery of novel human medicines to combat the looming problem of growing drug-resistance. For this to happen, however, it is imperative that we urgently prioritize the preservation of our wild forest pharmacies as well as our primate cousins who inhabit them.

### Materials availability

Voucher specimens for each species were deposited at the Makerere University Herbarium in Kampala, Uganda for taxonomic identification and storage. A duplicate set was deposited at the University of Oxford Herbarium for permanent storage.

## Supporting information

S1 Fig Budongo chimpanzees consuming resources tested in this study. a.) IN eating *K*. *anthotheca* bark and resin b.) MZ eating *S*. *myrtina* bark c.) KC stripping *A*. *polystachyus* pith d.) MB eating *C*. *patens* dead wood e.) OZ eating *S*. *guineense* bark (post-study period) g.) MZ eating *F*. *exasperata* bark.
(TIF)

S2 Fig Generalized multi-method workflow used in this study. (TIF)

S3 Fig Voucher samples collected in duplicate. *a*.*) C*. *alexandri* (00243133G) *b*.*) A*. *polystachius* (00243136J) *c*.*) W*. *elongata* (00243129L) *d*.*) C*. *parasitica* (00243122E) *e*.*) K*. *anthotheca* (00243123F) *f*.*) F*. *variifolia* (51195) *g*.*) M*. *leucantha* (51203) *h*.*) A*. *boonei* (51204) *i*.*) D*. *dewevrei* (00243132F) *j*.*) S*. *guineense* (00243135I) *k*.*) S*. *myrtina* (00243128K) *l*.*) F*. *exasperata* (00243130D).
(TIF)

S4 Fig Plate layouts for growth inhibition assays. [Top] **Library Screen:** done in 96-wells-mikrotiterplate; AB: Antibiotic as positive control; DMSO: vehicle control / negative control; GC: growth control: containing working culture, to check whether the bacterium grew/active; [Bottom] **Dose-Response:** done in descending concentration of samples, DMSO, and antibiotic. MB: Media blank, consisted of CAMHB as negative/ sterile media control; DMSO as negative/ vehicle control; GC: growth control, consisted of working culture.
(TIF)

S5 Fig ELISA assay setup for anti-inflammatory assay. (TIF)

S1 File Supplementary materials: *Methods*. (PDF)

S2 File Supplementary tables. (PDF)
