# Supplementary figures and images for: Pharmacological and behavioral investigation of putative self-medicative plants in Budongo chimpanzee diets
Source: PLoS One. 2024 Jun 20;19(6):e0305219. doi: 10.1371/journal.pone.0305219 (PMC11189245; doi:10.1371/journal.pone.0305219)

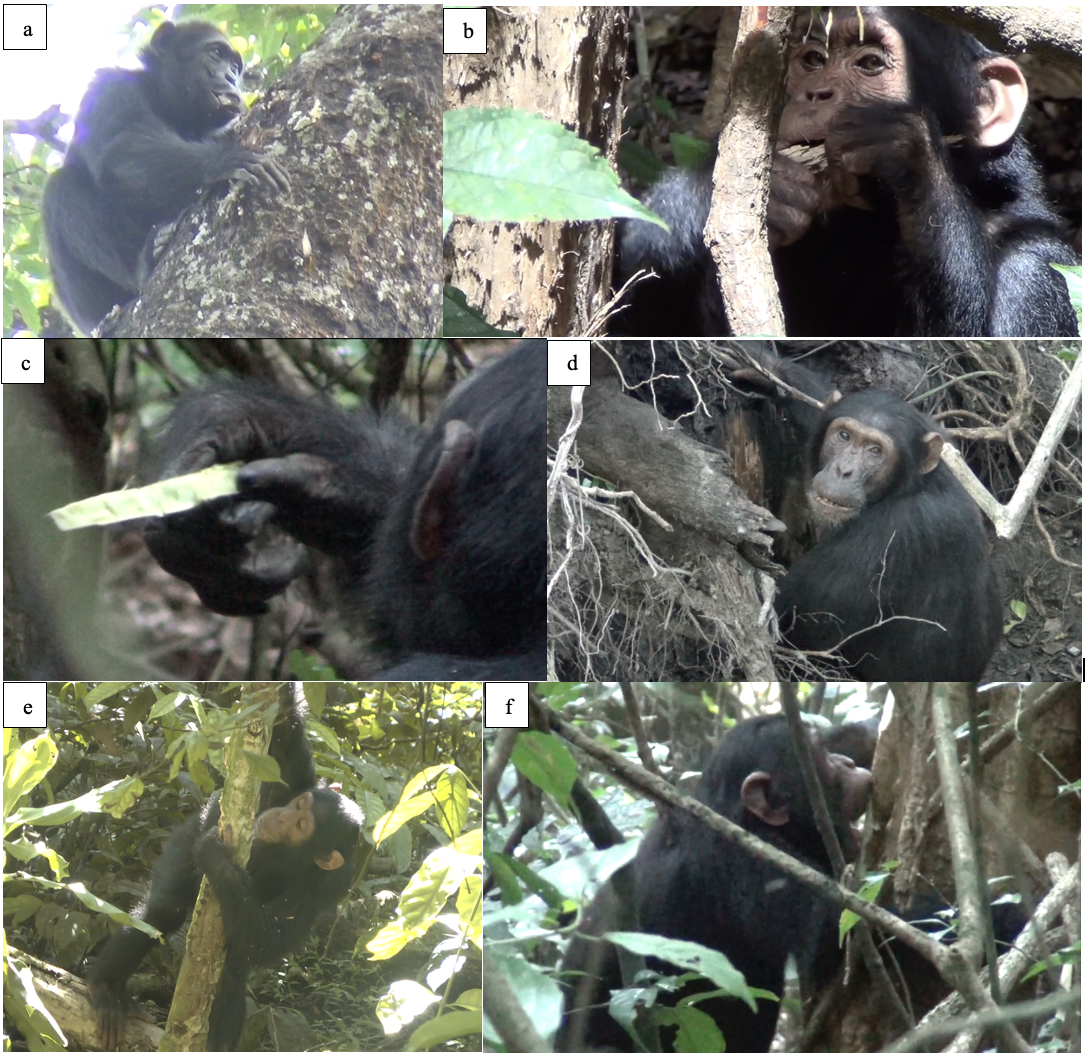

Supplement: S1 Fig — a.) IN eating K. anthotheca bark and resin b.) MZ eating S. myrtina bark c.) KC stripping A. polystachyus pith d.) MB eating C. patens dead wood e.) OZ eating S. guineense bark (post-study period) g.) MZ eating F. exasperata bark. (TIF) [file pone.0305219.s001.tif]

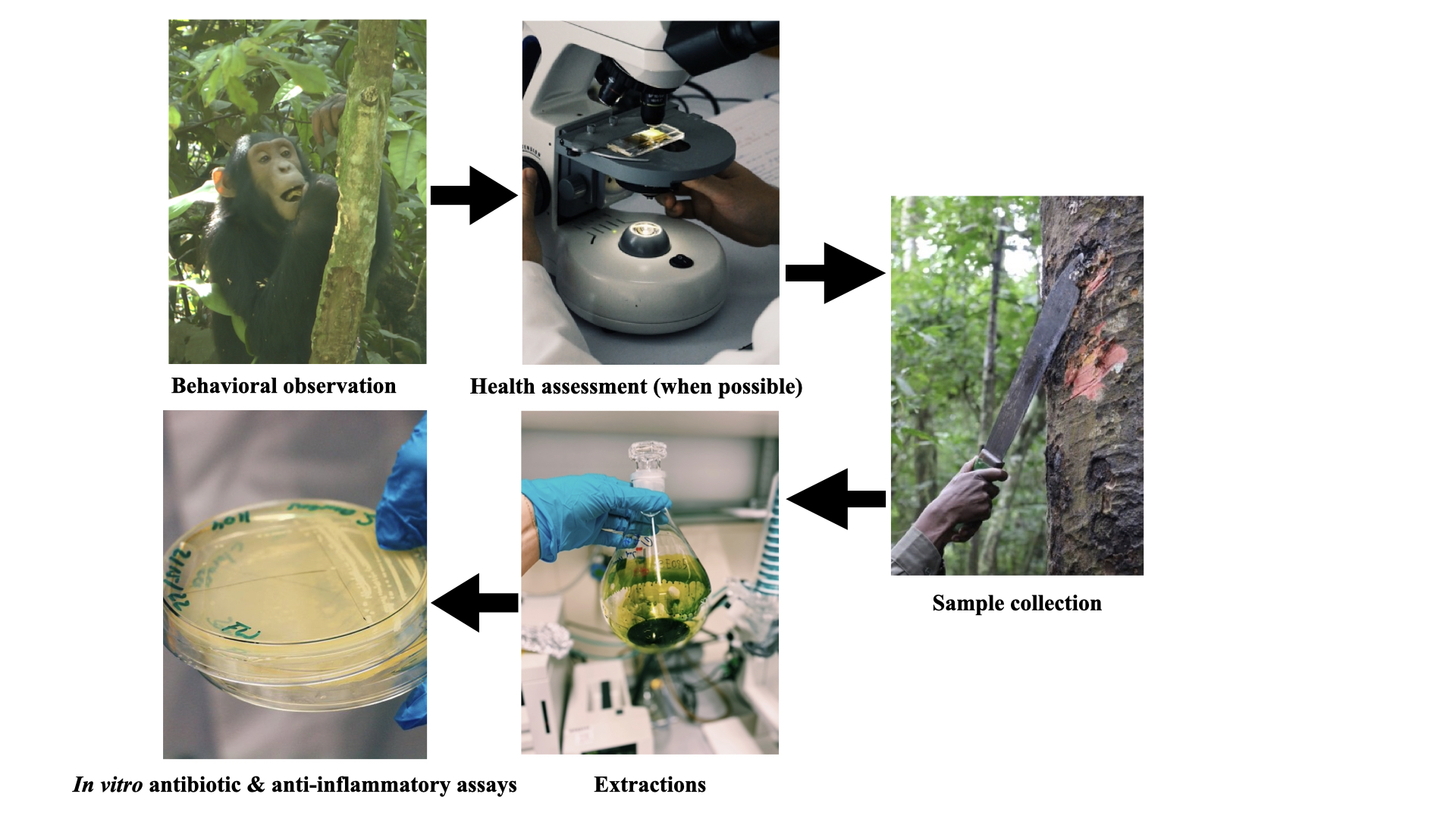

Supplement: S2 Fig — (TIF) [file pone.0305219.s002.tif]

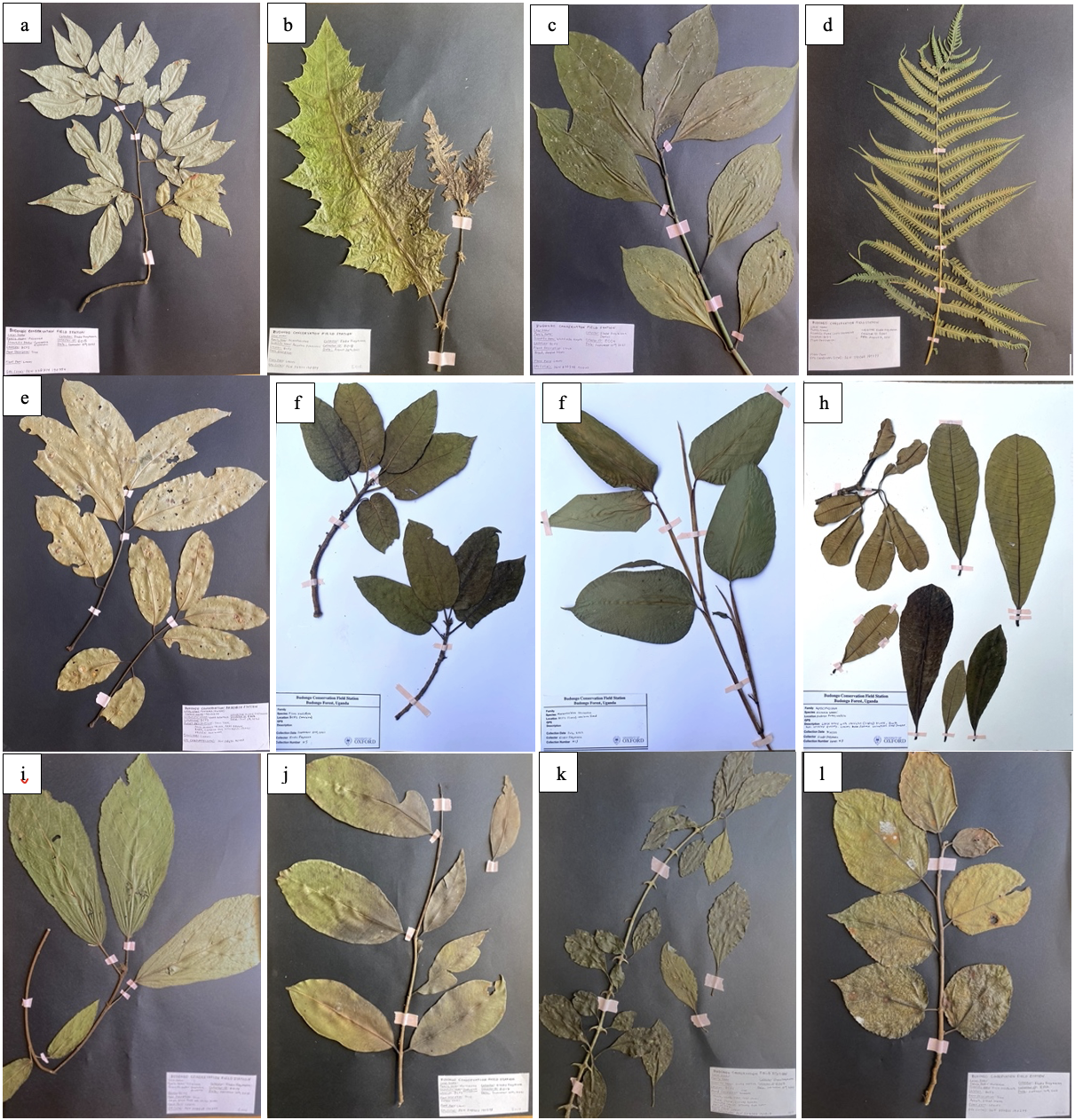

Supplement: S3 Fig — a.) C. alexandri (00243133G) b.) A. polystachius (00243136J) c.) W. elongata (00243129L) d.) C. parasitica (00243122E) e.) K. anthotheca (00243123F) f.) F. variifolia (51195) g.) M. leucantha (51203) h.) A. boonei (51204) i.) D. dewevrei (00243132F) j.) S. guineense (00243135I) k.) S. myrtina (00243128K) l.) F. exasperata (00243130D). (TIF) [file pone.0305219.s003.tif]

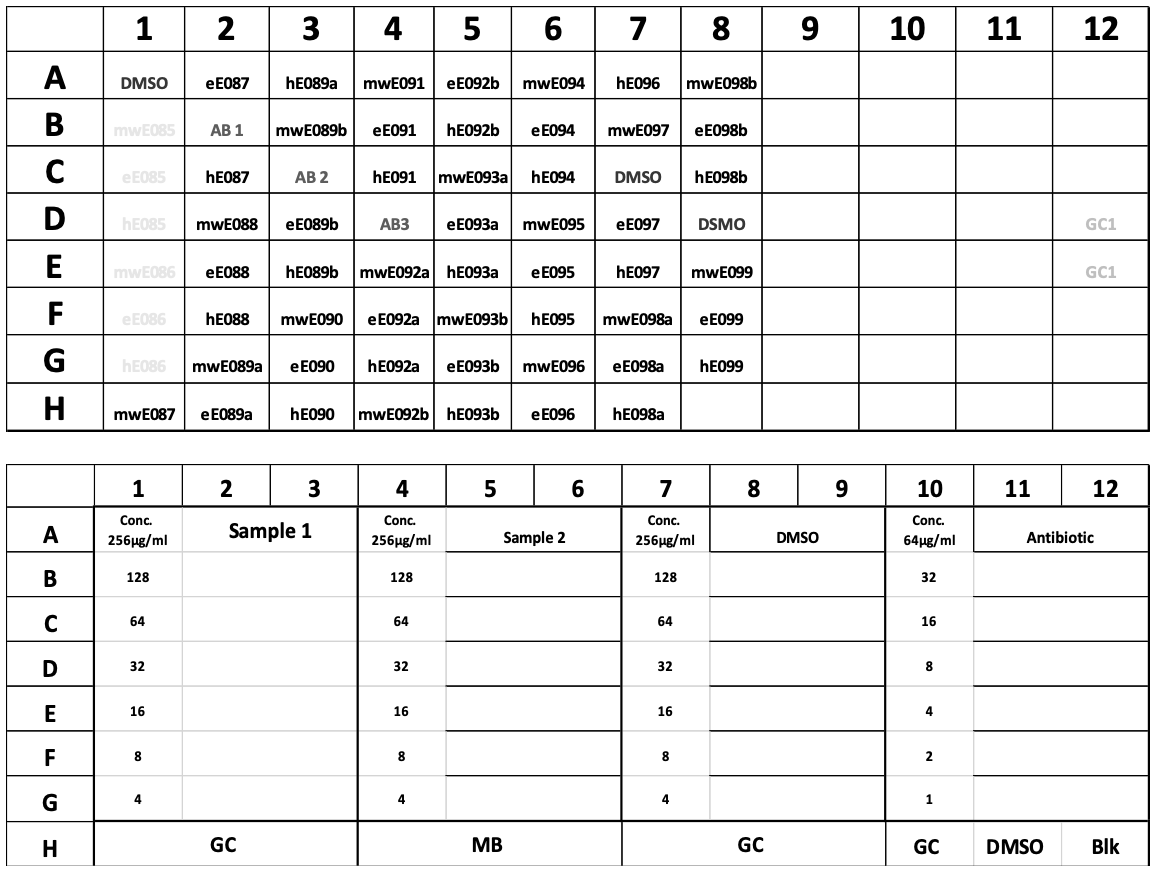

Supplement: S4 Fig — [Top] Library Screen: done in 96-wells-mikrotiterplate; AB: Antibiotic as positive control; DMSO: vehicle control / negative control; GC: growth control: containing working culture, to check whether the bacterium grew/active; [Bottom] Dose-Response: done in descending concentration of samples, DMSO, and antibiotic. MB: Media blank, consisted of CAMHB as negative/ sterile media control; DMSO as negative/ vehicle control; GC: growth control, consisted of working culture. (TIF) [file pone.0305219.s004.tif]

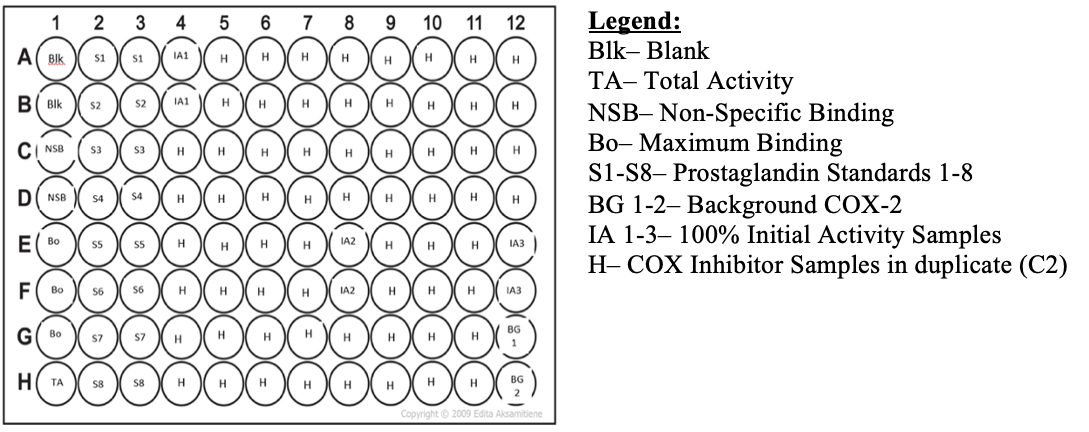

Supplement: S5 Fig — (TIF) [file pone.0305219.s005.tif]
