# Supplementary material for: Pharmacological and behavioral investigation of putative self-medicative plants in Budongo chimpanzee diets
Source: PLoS One. 2024 Jun 20;19(6):e0305219. doi: 10.1371/journal.pone.0305219 (PMC11189245; doi:10.1371/journal.pone.0305219)
Supplement: S1 File — (PDF) [file pone.0305219.s006.pdf]

Supplementary Information

Supplementary Methods: Collection of Sample Material (Additional Information)

When possible, bark samples were collected from trees with traces of previous bark stripping. In all but one case (*S. myrtina*), stem bark samples were cut directly from the trunk of the tree. For this sample, stripped bark refuse was taken from the base of the tree. After harvesting, plants were cut into small pieces and dried in the shade for two weeks. Samples were turned twice a day to prevent mold. Once dry, samples were transferred to paper bags and stored in a dark, dry room until export.

Supplementary Methods: Bacterial culture preparation and bacteria standardization

Methods for antibacterial assays used in this study have previously been described in Schultz et al. [124]. Strains were streaked from freezer stock, maintained on tryptic soy agar (TSA), and incubated overnight at 37°C. To prepare the working suspensions for the bioassays, 14 mL tryptic soy broth (TSB) were inoculated with a bacterial colony and incubated overnight at 37°C. Working suspensions were placed at an angle and were constantly shaking at 200 rpm. The bacterial suspension was standardized via determination of the overnight culture's optical density at 590 nm using a UV-vis spectrophotometer and to a confluence of $5x10^5$ CFU/mL, ensuring individual experiments were carried out using the same bacterial count. To create the standardized working culture, calculated quantities of assay growth media (cation-adjusted Mueller-Hinton broth (CAMHB)) and overnight culture were combined.
