# Supplementary material for: Pharmacological and behavioral investigation of putative self-medicative plants in Budongo chimpanzee diets
Source: PLoS One. 2024 Jun 20;19(6):e0305219. doi: 10.1371/journal.pone.0305219 (PMC11189245; doi:10.1371/journal.pone.0305219)
Supplement: S2 File — (PDF) [file pone.0305219.s007.pdf]

Table S1: Pre-screening results of the *in vitro* antibacterial trials at 256 µg/mL (library screen)

		Inhibition [%] ≥ 40										
**Plant species**	**Extract ID**	***S. aureus* DSM 1104**	***S. aureus* DSM 18827**	***A. baumannii* DSM 102929**	***E. cloacae* DSM 30054**	***K. pneumoniae* DSM 16609**	***P. aeruginosa* DSM 1117**	***E. faecium* DSM 13590**	***E. coli* DSM 498**	***E. coli* DSM 15076**	***S. maltophilia* DSM 50170**	***S. enterica* subsp. *enterica* DSM 11320**
C. parasitica	mwE087	-	-	-	-	-	-	-	-	-	-	-
	eE087	-	-	-	-	-	-	-	+	-	-	-
	hE087	-	-	-	-	-	-	-	+	+	-	-
K. anthotheca	mwE088	-	-	+	-	-	-	+	+	+	+	-
	eE088	-	-	-	-	-	-	+	+	+	-	-
	hE088	-	-	-	-	-	-	+	-	+	-	+
S. myrtina	mwE089a	-	-	-	-	-	-	-	+	+	-	-
	eE089a	-	-	-	-	-	-	+	+	+	-	-
	hE089a	-	-	-	-	-	-	-	+	-	-	-
	mwE089b	-	-	-	-	-	-	-	-	+	-	-
	eE089b	-	-	+	-	-	-	+	+	+	-	-
	hE089b	-	+	+	-	-	-	-	-	+	-	-
W. elongata	mwE090	-	-	-	-	-	-	-	-	+	-	-
	eE090	-	-	-	-	-	-	+	+	+	-	-
	hE090	-	-	-	-	-	-	-	-	-	-	-
C. patens	mwE091	-	-	-	-	-	-	-	-	+	-	-
	eE091	+	-	+	-	-	-	+	+	+	+	+
	hE091	-	-	-	-	-	-	+	-	+	-	-
A. boonei	mwE092a	-	-	-	-	-	-	-	-	+	-	-
	eE092a	-	-	-	-	-	-	-	-	-	-	-
	hE092a	-	-	+	-	-	-	-	-	-	-	-
	mwE092b	-	-	-	-	-	-	-	+	+	-	-
	eE092b	+	-	+	-	-	-	+	+	+	-	-
	hE092b	+	+	-	-	-	-	+	+	+	-	+
F. exasperata	mwE093a	-	-	-	-	-	-	-	-	+	-	-
	eE093a	-	-	-	-	-	-	+	+	+	-	-
	hE093a	-	-	-	-	-	-	+	-	-	-	-
	mwE093b	-	-	-	-	-	-	-	-	+	-	-
	eE093b	-	-	-	-	-	-	-	-	-	-	-
	hE093b	-	-	-	-	-	-	-	+	+	-	-
M. leucantha	mwE094	-	-	-	-	-	-	-	-	+	-	-
	eE094	-	-	-	-	-	-	-	+	+	-	-
	hE094	-	-	+	-	-	-	-	-	+	-	-
D. dewevrei	mwE095	-	-	+	-	-	-	-	+	+	+	-
	eE095	-	-	-	-	-	-	-	+	+	-	-
	hE095	-	-	-	-	-	-	-	-	+	-	-
C. alexandri	mwE096	+	-	+	-	-	-	-	-	+	+	-
	eE096	-	-	-	-	-	-	-	+	+	-	-
	hE096	-	-	-	-	-	-	-	+	+	-	-
F. variifolia	mwE097	-	-	-	-	-	-	-	-	-	-	-
	eE097	-	-	-	-	-	-	+	+	+	-	-
	hE097	-	-	-	-	-	-	-	+	+	-	-
S. guineense	mwE098a	+	+	+	+	-	+	-	+	+	+	-
	eE098a	-	+	+	+	-	-	-	+	+	+	-
	hE098a	-	-	-	-	-	-	-	-	+	-	-
	mwE098b	+	+	+	+	-	-	-	+	+	+	-
	eE098b	-	+	+	+	-	-	-	-	+	+	-
	hE098b	-	-	+	+	-	-	-	+	+	+	-
A. polystachius	mwE099	-	-	-	-	-	-	-	-	-	-	-
	eE099	-	-	-	-	-	-	+	+	+	-	-
	hE099	+	-	+	-	-	-	+	+	+	-	-

\+ : samples with ≥40% Inhibition
\- : samples with <40% Inhibition

Table S2: IC_{50} and MIC values with standard deviations obtained from *in vitro* dose-response assays

	Gram	+				+				-				-				-			
**Scientific name**	**Extract ID**	***S. aureus* DSM 1104**				***S. aureus* DSM 18827**				***A. baumannii* DSM 102929**				***E. cloacae* DSM 30054**				***P. aeruginosa* DSM 1117**			
		**IC_{50}**	s	**MIC**	s	**IC_{50}**	s	**IC_{50}**	s	**IC_{50}**	s	**MIC**	s	**IC_{50}**	s	**MIC**	s	**IC_{50}**	s	**MIC**	s
C. parasitica	eE087	-	-	-	-	-	-	-	-	-	-	-	-	-	-	-	-	-	-	-	-
	hE087	-	-	-	-	-	-	-	-	-	-	-	-	-	-	-	-	-	-	-	-
K. anthotheca	mwE088	-	-	-	-	-	-	-	-	>256	-	>256	-	-	-	-	-	-	-	-	-
	eE088	-	-	-	-	-	-	-	-	-	-	-	-	-	-	-	-	-	-	-	-
	hE088	-	-	-	-	-	-	-	-	-	-	-	-	-	-	-	-	-	-	-	-
S. myrtina	mwE089a	-	-	-	-	-	-	-	-	-	-	-	-	-	-	-	-	-	-	-	-
	eE089a	-	-	-	-	-	-	-	-	-	-	-	-	-	-	-	-	-	-	-	-
	hE089a	-	-	-	-	-	-	-	-	-	-	-	-	-	-	-	-	-	-	-	-
	mwE089b	-	-	-	-	-	-	-	-	-	-	-	-	-	-	-	-	-	-	-	-
	eE089b	-	-	-	-	-	-	-	-	>256	-	>256	-	-	-	-	-	-	-	-	-
	hE089b	-	-	-	-	>256	-	>256	-	256	0.9	>256	-	-	-	-	-	-	-	-	-
W. elongata	mwE090	-	-	-	-	-	-	-	-	-	-	-	-	-	-	-	-	-	-	-	-
	eE090	-	-	-	-	-	-	-	-	-	-	-	-	-	-	-	-	-	-	-	-
C. patens	mwE091	-	-	-	-	-	-	-	-	-	-	-	-	-	-	-	-	-	-	-	-
	eE091	128	0.9	256	0.4	-	-	-	-	>256	-	>256	-	-	-	-	-	-	-	-	-
	hE091	-	-	-	-	-	-	-	-	-	-	-	-	-	-	-	-	-	-	-	-
A. boonei	mwE092a	-	-	-	-	-	-	-	-	-	-	-	-	-	-	-	-	-	-	-	-
	hE092a	-	-	-	-	-	-	-	-	>256	-	>256	-	-	-	-	-	-	-	-	-
	mwE092b	-	-	-	-	-	-	-	-	-	-	-	-	-	-	-	-	-	-	-	-
	eE092b	32	4.3	128	0.1	-	-	-	-	>256	-	>256	-	-	-	-	-	-	-	-	-
	hE092b	16	9.5	32	0.1	32	2.5	>256	-	-	-	-	-	-	-	-	-	-	-	-	-
F. exasperata	mwE093a	-	-	-	-	-	-	-	-	-	-	-	-	-	-	-	-	-	-	-	-
	eE093a	-	-	-	-	-	-	-	-	-	-	-	-	-	-	-	-	-	-	-	-
	hE093a	-	-	-	-	-	-	-	-	-	-	-	-	-	-	-	-	-	-	-	-
	mwE093b	-	-	-	-	-	-	-	-	-	-	-	-	-	-	-	-	-	-	-	-
	hE093b	-	-	-	-	-	-	-	-	-	-	-	-	-	-	-	-	-	-	-	-
M. leucantha	mwE094	-	-	-	-	-	-	-	-	-	-	-	-	-	-	-	-	-	-	-	-
	eE094	-	-	-	-	-	-	-	-	>256	-	>256	-	-	-	-	-	-	-	-	-
	hE094	-	-	-	-	-	-	-	-	-	-	-	-	-	-	-	-	-	-	-	-
D. dewevrei	mwE095	-	-	-	-	-	-	-	-	>256	-	>256	-	-	-	-	-	-	-	-	-
	eE095	-	-	-	-	-	-	-	-	-	-	-	-	-	-	-	-	-	-	-	-
	hE095	-	-	-	-	-	-	-	-	-	-	-	-	-	-	-	-	-	-	-	-
C. alexandri	mwE096	>256	-	>256	-	-	-	-	-	>256	-	>256	-	-	-	-	-	-	-	-	-
	eE096	-	-	-	-	-	-	-	-	-	-	-	-	-	-	-	-	-	-	-	-
	hE096	-	-	-	-	-	-	-	-	-	-	-	-	-	-	-	-	-	-	-	-
F. variifolia	eE097	-	-	-	-	-	-	-	-	-	-	-	-	-	-	-	-	-	-	-	-
	hE097	-	-	-	-	-	-	-	-	-	-	-	-	-	-	-	-	-	-	-	-
S. guineense	mwE098a	>256	-	>256	-	>256	-	>256	-	64	1.1	>256	-	128	0.6	>256	-	64	1.9	>256	-
	eE098a	-	-	-	-	>256	-	>256	-	128	0.7	>256	-	256	4.2	>256	-	-	-	-	-
	hE098a	-	-	-	-	-	-	-	-	-	-	-	-	-	-	-	-	-	-	-	-
	mwE098b	128	1.1	>256	-	>256	-	>256	-	128	0.4	>256	-	128	0.2	>256	-	-	-	-	-
	eE098b	-	-	-	-	>256	-	>256	-	>256	-	>256	-	256	2.6	>256		-	-	-	-
	hE098b	-	-	-	-	>256	-	>256	-	>256	-	>256	-	>256	-	>256	-	-	-	-	-
A. polystachius	eE099	-	-	-	-	-	-	-	-	-	-	-	-	-	-	-	-	-	-	-	-
	hE099	256	4.4	>256	-	-	-	-	-	-	-	-	-	-	-	-	-	-	-	-	-
**Vancomycin**		<1	-	2	0	<1	-	1	1.8	>64	-	>64	-	>64	-	>64	-	>64	-	>64	-
**Gentamicin**		0.125		0.25		0.5	0.9	1	0.1	>64	-	>64	-	n.t.				n.t.			
**Ciprofloxacin**		n.t.				n.t.				>64	-	>64	-	n.t.				n.t.			
**Tetracyclin**		n.t.				n.t.				1	9.4	2	1	n.t.				n.t.			
**Chloramphenicol**		2	0.6	8	0.3	4	1.1	16	0.8	32	1.3	64	0.9	2	1.7	4	8.1	32	0.3	>64	-

NB: Only extracts showing growth inhibition ≥40% in the library screen at 256 µg/mL are listed. IC_{50} and MIC values are expressed as concentration (µg/mL).
s: standard deviation. **n.t:** not tested

Table S2: Continued

	Gram	+				-				-				-				-			
**Scientific name**	**Extract ID**	***E. faecium* DSM 13590**				***E. coli* DSM 498**				***E. coli* DSM 1576**				***S. maltophilia* DSM 50170**				***S. enterica* subsp. *enterica DSM 11320***			
		**IC_{50}**	s	**MIC**	s	**IC_{50}**	s	**MIC**	s	**IC_{50}**	s	**MIC**	s	**IC_{50}**	s	**MIC**	s	**IC_{50}**	s	**MIC**	s
C. parasitica	eE087	-	-	-	-	>256	-	>256	-	-	-	-	-	-	-	-	-	-	-	-	-
	hE087	-	-	-	-	>256	-	>256	-	128	0.5	>256	-	-	-	-	-	-	-	-	-
K. anthotheca	mwE088	16	3.7	32	2.3	>256	-	>256	-	16	1.4	256	0.3	64	4.4	>256	-	-	-	-	-
	eE088	64	1.8	64	1.8	>256	-	>256	-	64	0.9	>256	-	-	-	-	-	-	-	-	-
	hE088	64	8.4	128	5	-	-	-	-	64	5.7	>256	-	-	-	-	-	256	5.5	>256	-
S. myrtina	mwE089a	-	-	-	-	>256	-	>256	-	128	2.8	>256	-	-	-	-	-	-	-	-	-
	eE089a	64	2.1	>256	-	>256	-	>256	-	256	2.1	>256	-	-	-	-	-	-	-	-	-
	hE089a	-	-	-	-	>256	-	>256	-	-	-	-	-	-	-	-	-	-	-	-	-
	mwE089b	-	-	-	-	-	-	-	-	256	0.2	>256	-	-	-	-	-	-	-	-	-
	eE089b	128	5.8	>256	-	>256	-	>256	-	256	0.8	>256	-	-	-	-	-	-	-	-	-
	hE089b	-	-	-	-	-	-	-	-	256	1.3	>256	-	-	-	-	-	-	-	-	-
W. elongata	mwE090	-	-	-	-	-	-	-	-	128	0.3	>256	-	-	-	-	-	-	-	-	-
	eE090	64	1.3	128	3.4	>256	-	>256	-	256	1.4	>256	-	-	-	-	-	-	-	-	-
C. patens	mwE091	-	-	-	-	-	-	-	-	128	2.2	>256	-	-	-	-	-	-	-	-	-
	eE091	64	0.9	64	0.9	>256	-	>256	-	128	18.1	>256	-	256	0.7	>256	-	>256	-	>256	-
	hE091	64	2.2	>256	-	-	-	-	-	128	2.2	>256	-	-	-	-	-	-	-	-	-
A. boonei	mwE092a	-	-	-	-	-	-	-	-	128	1.3	>256	-	-	-	-	-	-	-	-	-
	hE092a	-	-	-	-	-	-	-	-	-	-	-	-	-	-	-	-	-	-	-	-
	mwE092b	-	-	-	-	256	2.1	>256	-	32	3.1	>256	-	-	-	-	-	-	-	-	-
	eE092b	16	1.2	64	1.6	>256	-	>256	-	128	8.9	>256	-	-	-	-	-	-	-	-	-
	hE092b	16	3.1	>256	-	>256	-	>256	-	256	0.4	>256	-	-	-	-	-	256	5.3	256	5.3
F. exasperata	mwE093a	-	-	-	-	-	-	-	-	128	1.4	>256	-	-	-	-	-	-	-	-	-
	eE093a	32	0.1	128	7.5	>256	-	>256	-	128	3.0	>256	-	-	-	-	-	-	-	-	-
	hE093a	32	4.1	>256	-	-	-	-	-	-	-	-	-	-	-	-	-	-	-	-	-
	mwE093b	-	-	-	-	-	-	-	-	256	1.0	>256	-	-	-	-	-	-	-	-	-
	hE093b	-	-	-	-	>256	-	>256	-	256	2.2	>256	-	-	-	-	-	-	-	-	-
M. leucantha	mwE094	-	-	-	-	-	-	-	-	128	5.0	-	-	-	-	-	-	-	-	-	-
	eE094	-	-	-	-	>256	-	>256	-	256	3.3	>256	-	-	-	-	-	-	-	-	-
	hE094	-	-	-	-	-	-	-	-	256	1.4	>256	-	-	-	-	-	-	-	-	-
D. dewevrei	mwE095	-	-	-	-	>256	-	>256	-	256	2.1	>256	-	256	0.8	>256	-	-	-	-	-
	eE095	-	-	-	-	>256	-	>256	-	256	3.4	>256	-	-	-	-	-	-	-	-	-
	hE095	-	-	-	-	-	-	-	-	>256	-	>256	-	-	-	-	-	-	-	-	-
C. alexandri	mwE096	-	-	-	-	-	-	-	-	256	0.6	>256	-	256	2.2	>256	-	-	-	-	-
	eE096	-	-	-	-	>256	-	>256	-	256	3.9	>256	-	-	-	-	-	-	-	-	-
	hE096	-	-	-	-	>256	-	>256	-	256	1.9	>256	-	-	-	-	-	-	-	-	-
F. variifolia	eE097	64	2.5	>256	-	>256	-	>256	-	256	0.4	>256	-	-	-	-	-	-	-	-	-
	hE097	-	-	-	-	>256	-	>256	-	256	0.6	>256	-	-	-	-	-	-	-	-	-
S. guineense	mwE098a	-	-	-	-	>256	-	>256	-	64	2.7	>256	-	32	0.3	256	1.0	-	-	-	-
	eE098a	-	-	-	-	>256	-	>256	-	128	4.7	>256	-	64	0.8	256	4.1	-	-	-	-
	hE098a	-	-	-	-	-	-	-	-	128	1.4	>256	-	-	-	-	-	-	-	-	-
	mwE098b	-	-	-	-	128	0.2	>256	-	32	5.1	128	0.7	32	0.5	>256	-	-	-	-	-
	eE098b	-	-	-	-	>256	-	>256	-	128	2.6	256	2	128	2.7	>256	-	-	-	-	-
	hE098b	-	-	-	-	-	-	-	-	256	1	>256	-	128	0.8	>256	-	-	-	-	-
A. polystachius	eE099	128	7.5	256	7.1	>256	-	>256	-	256	1.5	>256	-	-	-	-	-	-	-	-	-
	hE099	32	7.6	128	7.9	>256	-	>256	-	256	1.0	>256	-	-	-	-	-	-	-	-	-
**Vancomycin**		>64	-	>64	-	n.t.				n.t.				n.t.				n.t.			
**Gentamicin**		8	2.3	>64	-	<1	-	1	3.9	n.t.				8	0.3	8	0.3	n.t.			
**Ciprofloxacin**		n.t.				n.t.				n.t.				n.t.				n.t.			
**Tetracyclin**		n.t.				<1	-	2	0.5	n.t.				n.t.				1	1.4	2	0.4
**Chloramphenicol**		-	2	4	0.8	n.t.				<1	-	4	0.1	n.t.				4	6.8	8	5.5

NB: Only extracts showing growth inhibition ≥40% in the library screen at 256 µg/mL are listed. IC_{50} and MIC values are expressed as concentration (µg/mL).
s: standard deviation. **n.t:** not tested

Table S3: Summary of COX-2 and antibacterial results across species

			LIBRARY SCREEN ACTIVITY:				≤256μg/mL
**Plant Species**	**Plant Part**	**Extract ID**	**COX-2 [50μg/mL]**	**Antibacterial Library Screen**	**Number of active extracts on antibacterial library screen**	**Number of strains that extracts were active against on library screen**	**Number of strains each extract was active against on dose-response assays (≤256μg/mL)**
C. parasitica	whole plant	mwE087	+	-	2	0	0
		eE087	+	+		1	nt
		hE087	+	+		2	1
K. anthotheca	bark	mwE088	+	+	3	5	3
		eE088	+	+		3	2
		hE088	+	+		3	3
S. myrtina	dead wood	mwE089a	-	+	6	2	1
		eE089a	+	+		3	2
		hE089a	+	+		1	0
	stripped wood	mwE089b	+	+		1	1
		eE089b	+	+		4	2
		hE089b	-	+		3	2
W. elongata	leaves	mwE090	+	+	2	1	1
		eE090	+	+		3	2
		hE090	+	-		0	nt
C. patens	dead wood	mwE091	+	+	3	1	1
		eE091	+	+		7	4
		hE091	-	+		2	2
A. boonei	bark	mwE092a	-	+	5	1	1
		eE092a	-	-		0	nt
		hE092a	+	+		1	0
	dead wood	mwE092b	+	+		2	2
		eE092b	+	+		5	3
		hE092b	+	+		6	5
F. exasperata	bark	mwE093a	+	+	5	1	1
		eE093a	+	+		3	2
		hE093a	+	+		1	1
	leaves	mwE093b	+	+		1	1
		eE093b	+	-		0	nt
		hE093b	+	+		2	1
M. leucantha	pith	mwE094	+	+	3	1	1
		eE094	+	+		2	1
		hE094	+	+		2	1
D. dewevrei	bark	mwE095	+	+	3	4	2
		eE095	+	+		2	1
		hE095	+	+		1	0
C. alexandri	bark	mwE096	-	+	3	4	2
		eE096	-	+		2	1
		hE096	+	+		2	1
F. variifolia	bark	mwE097	+	-	2	0	nt
		eE097	+	+		3	2
		hE097	+	+		2	1
S. guineense	bark	mwE098a	+	+	6	8	5
		eE098a	+	+		6	4
		hE098a	+	+		1	1
	leaves	mwE098b	-	+		7	6
		eE098b	+	+		5	3
		hE098b	+	+		5	2
A. polystachius	pith	mwE099	+	-	2	0	nt
		eE099	+	+		3	2
		hE099	+	+		5	3
		**Total**	43	45			

Table S4: Results from the COX-2 inhibition library screen at descending concentrations

				COX-2 Inhibition ≥50 %		
**Extract ID**	**Plant Species**	**Plant Part**	**Type of Extract**	**50 µg/ml**	**10 µg/ml**	**5 µg/ml**
mwE087	*C. parasitica*	whole plant	methanol/water (9:1, *v/v*)	+	+	+
eE087			ethyl acetate	+	+	+
hE087			*n*-hexane	+	+	+
mwE088	*K. anthotheca*	stem bark and resin	methanol/water (9:1, *v/v*)	+	+	+
eE088			ethyl acetate	+	+	+
hE088			*n*-hexane	+	+	+
mwE089a	*S. myrtina*	strips of stem bark (refuse)	methanol/water (9:1, *v/v*)	-	-	-
eE089a			ethyl acetate	+	-	-
hE089a			*n*-hexane	+	+	+
mwE089b		stem bark	methanol/water (9:1, *v/v*)	+	-	-
eE089b			ethyl acetate	+	+	-
hE089b			*n*-hexane	-	-	-
mwE090	*W. elongata*	leaves	methanol/water (9:1, *v/v*)	+	-	-
eE090			ethyl acetate	+	-	-
hE090			*n*-hexane	+	-	-
mwE091	*C. patens*	dead wood	methanol/water (9:1, *v/v*)	+	-	-
eE091			ethyl acetate	+	-	-
hE091			*n*-hexane	-	-	-
mwE092a	*A. boonei*	stem bark	methanol/water (9:1, *v/v*)	-	-	-
eE092a			ethyl acetate	-	-	-
hE092a			*n*-hexane	+	-	-
mwE092b		dead wood	methanol/water (9:1, *v/v*)	+	-	-
eE092b			ethyl acetate	+	-	-
hE092b			*n*-hexane	+	+	+
mwE093a	*F. exasperata*	stem bark	methanol/water (9:1, *v/v*)	+	-	-
eE093a			ethyl acetate	+	+	+
hE093a			*n*-hexane	+	+	+
mwE093b		leaves	methanol/water (9:1, *v/v*)	+	-	-
eE093b			ethyl acetate	+	+	-
hE093b			*n*-hexane	+	+	-
mwE094	*M. leucantha*	pith	methanol/water (9:1, *v/v*)	+	-	-
eE094			ethyl acetate	+	-	-
hE094			*n*-hexane	+	+	+
mwE095	*D. dewevrei*	stem bark	methanol/water (9:1, *v/v*)	+	-	-
eE095			ethyl acetate	+	-	-
hE095			*n*-hexane	+	-	-
mwE096	*C. alexandri*	stem bark	methanol/water (9:1, *v/v*)	-	-	-
eE096			ethyl acetate	-	-	-
hE096			*n*-hexane	+	+	+
mwE097	*F. variifolia*	stem bark	methanol/water (9:1, *v/v*)	+	-	-
eE097			ethyl acetate	+	+	+
hE097			*n*-hexane	+	+	+
mwE098a	*S. guineense*	stem bark	methanol/water (9:1, *v/v*)	+	-	-
eE098a			ethyl acetate	+	-	-
hE098a			*n*-hexane	+	+	+
mwE098b		leaves	methanol/water (9:1, *v/v*)	-	-	-
eE098b			ethyl acetate	+	-	-
hE098b			*n*-hexane	+	-	-
mwE099	*A. polystachyus*	pith	methanol/water (9:1, *v/v*)	+	-	-
eE099			ethyl acetate	+	+	+
hE099			*n*-hexane	+	+	+

Table S5: Information on Strain ID for each bacteria

Scientific Name	Strain ID	Resistances	Incubation length (h)	Type of isolate
Acinetobacter baumannii	DSM 102929	Penicillin G, Oxacillin, Ampicillin, Ticarcillin, Mezlocillin, Cefazolin, Cefotaxime, Vancomycin, Gentamicin, Ciprofloxacin	22	clinical isolate: human, skin, thigh
Enterobacter cloacae	DSM 30054	Penicillin G, Oxacillin, Ampicillin, Cefazolin	18	clinical isolate: spinal fluid
Enterococcus faecium	DSM 13590	Penicillin G, Oxacillin, Ticarcillin, Cefazolin, Cefotaxime, Aztreonam	18	clinical isolate: human faeces, rectal swab
Escherichia coli K12	DSM 498	Penicillin G, Oxacillin	18	unknown
Escherichia coli	DSM 1576	Oxacillin, Vancomycin, Lincomycin, Bacitracin, Clindamycin, Linezolid, Nystatin, Quinupristin/Dalfopristin, Teicoplanin	18	human faeces
Klebsiella pneumoniae	DSM 16609	Penicillin G, Oxacillin, Ampicillin, Ticarcillin, Mezlocillin	18	blood
Pseudomonas aeruginosa	DSM 1117	Chloramphenicol, Vancomycin, Penicillin G, Oxacillin, Ampicillin, Cefazolin	18	clinical isolate: blood culture; Human blood
Staphylococcus aureus	DSM 1104	Aztreonam	18	clinical isolate: Human
Staphylococcus aureus	DSM 18827	Penicillin G, Oxacillin, Cefazolin, Cefotaxime, Aztreonam, Imipenem	18	clinical isolate: tracheal secret; 65-year-old male
Stenotrophomonas maltophilia	DSM 50170 [ATTC: 13637]	Penicillin G, Oxacillin, Ampicillin, Ticarcillin, Cefalotin, Cefazolin, Aztreonam	18	clinical isolate: Oropharyngeal region of patient with mouth cancer
Salmonella enterica subsp. *enterica*	DSM 11320	Wildtype	18	unknown

NB: Permanent culture created in 2020; Source: Bacdive.dsmz.de and lab data

Table S6: List of antibiotics used

**Abbreviations**	**Antibacterial agents**
CIP	ciprofloxacin
TET	tetracycline
GEN	gentamicin
VAN	vancomycin
CHL	chloramphenicol
